# Supplementary figures and images for: A heterotrimeric complex of Toxoplasma proteins promotes parasite survival in interferon gamma-stimulated human cells
Source: PLoS Biol. 2023 Jul 17;21(7):e3002202. doi: 10.1371/journal.pbio.3002202 (PMC10373997; doi:10.1371/journal.pbio.3002202)

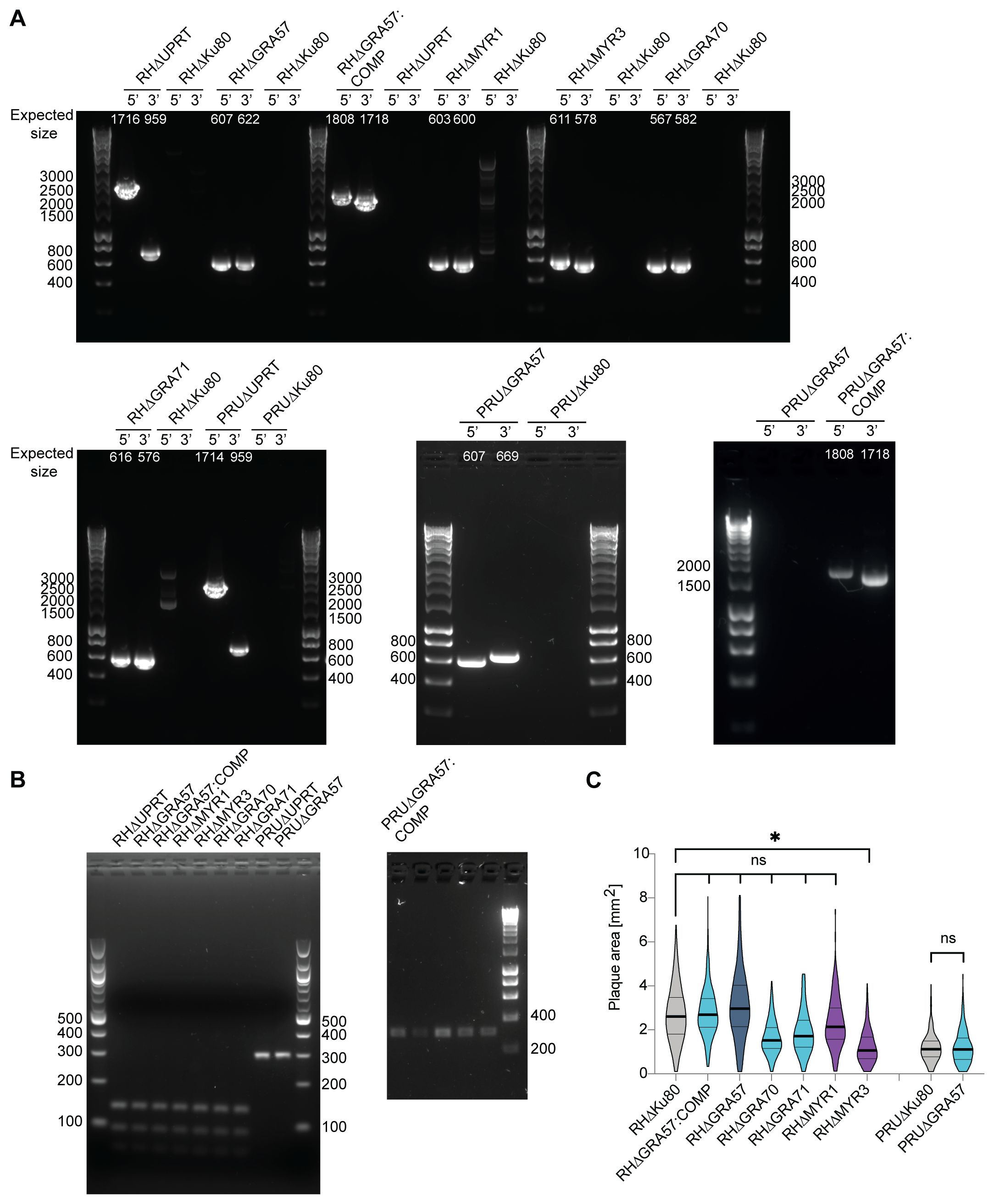

Supplement: S1 Fig — (A) PCR verification of successful integration of repair cassettes at indicated gene loci. Primers used for verification are listed in S11 Data. (B) Strain genotype verification by restriction fragment length polymorphism (RFLP) of the SAG3 gene. (C) Quantification of plaque area after 10 days growth in HFFs. Results are shown as violin plots with median and quartiles from a minimum of 3 biological replicates. p-values were calculated by one-way analysis of variance (ANOVA) with Tukey’s multiple comparison test. *, p < 0.05; ns, not significant. Source data for C can be found in S2 Data. (TIF) [file pbio.3002202.s001.tif]

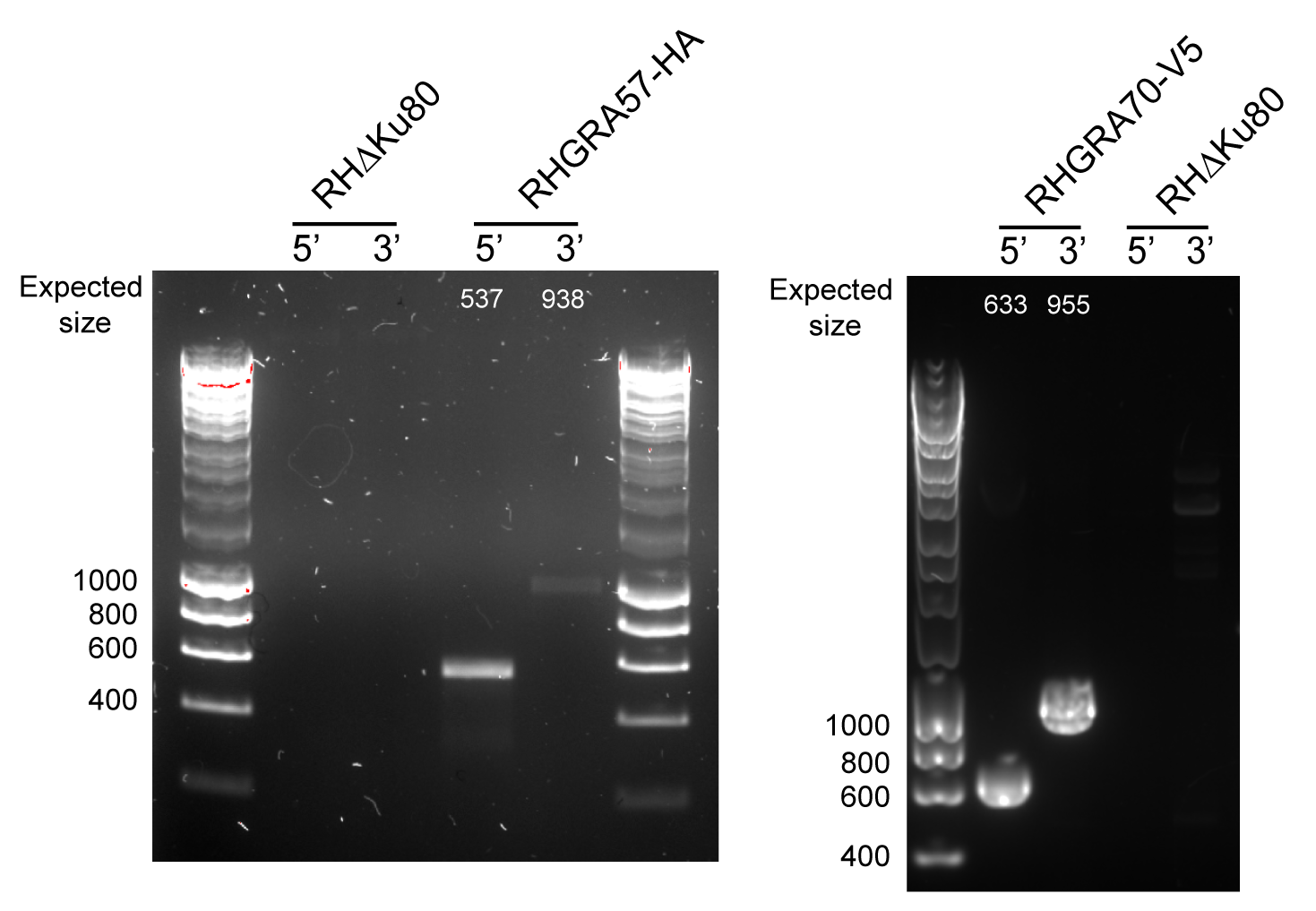

Supplement: S2 Fig — Primers used for verification are listed in S11 Data. (TIF) [file pbio.3002202.s002.tif]

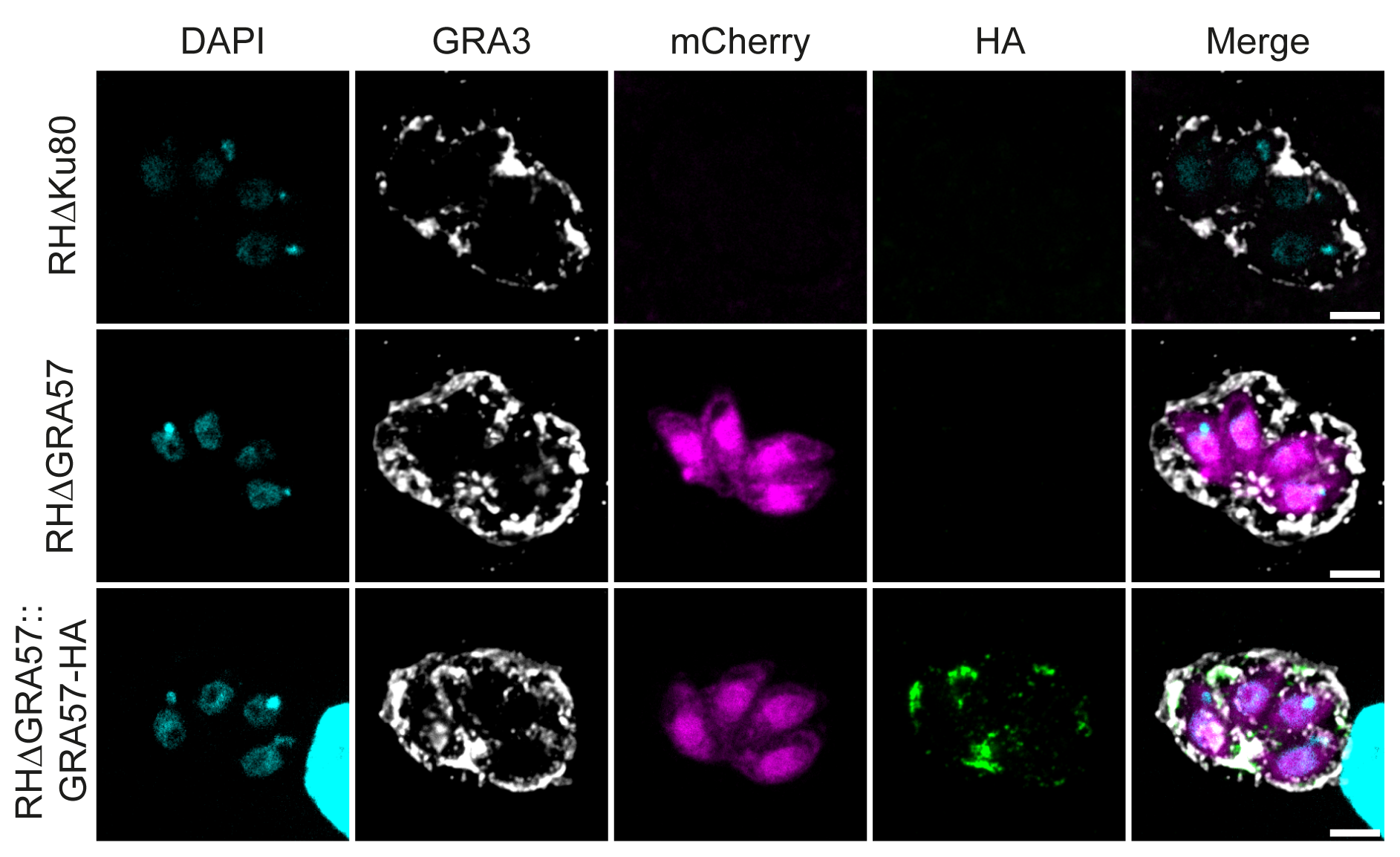

Supplement: S3 Fig — Scale bar represents 3 μm. (TIF) [file pbio.3002202.s003.tif]

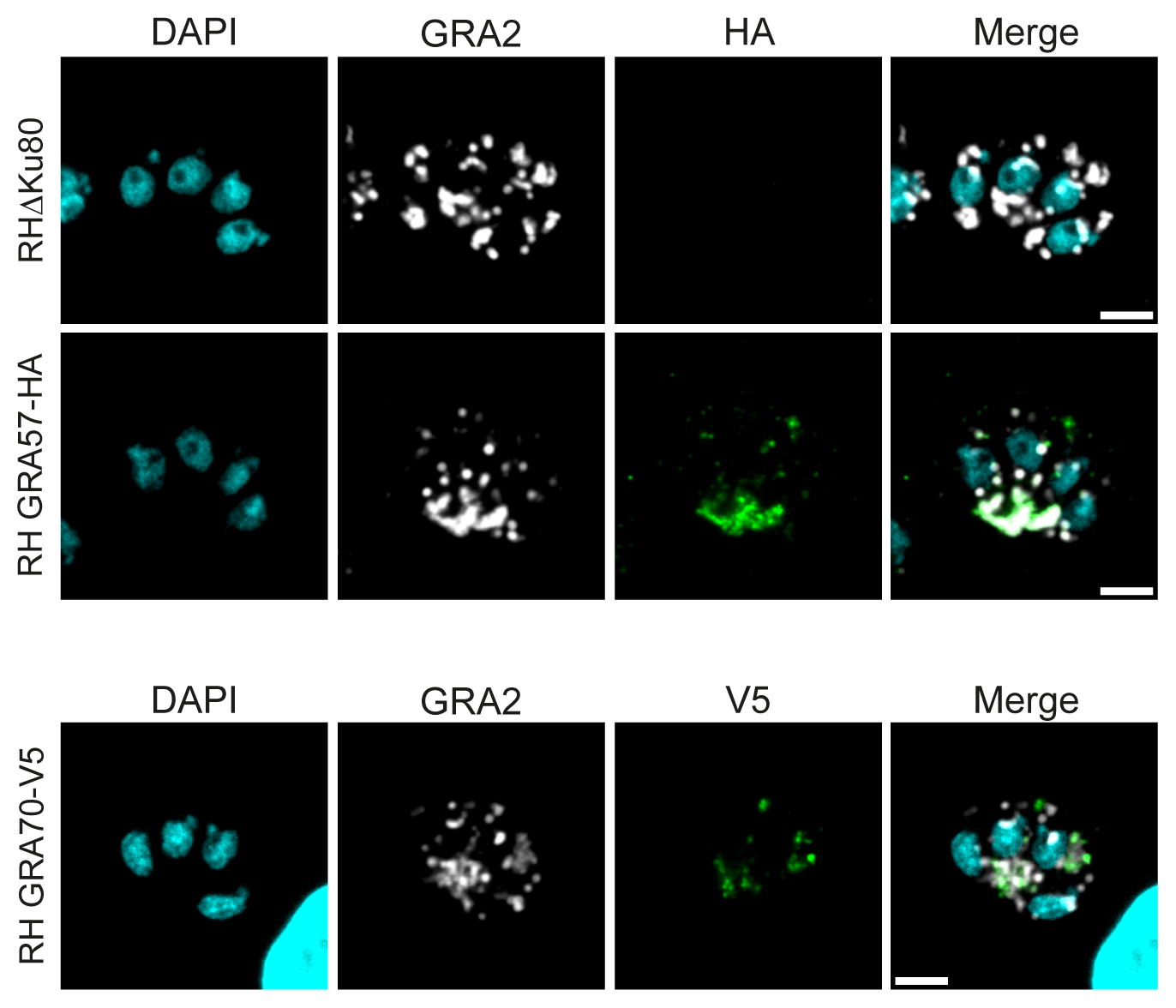

Supplement: S4 Fig — HFFs were pre-stimulated with 100 U/ml IFNγ for 24 h prior to infection, fixation, and staining as in Fig 3F. Scale bar = 3 μm. (TIF) [file pbio.3002202.s004.tif]

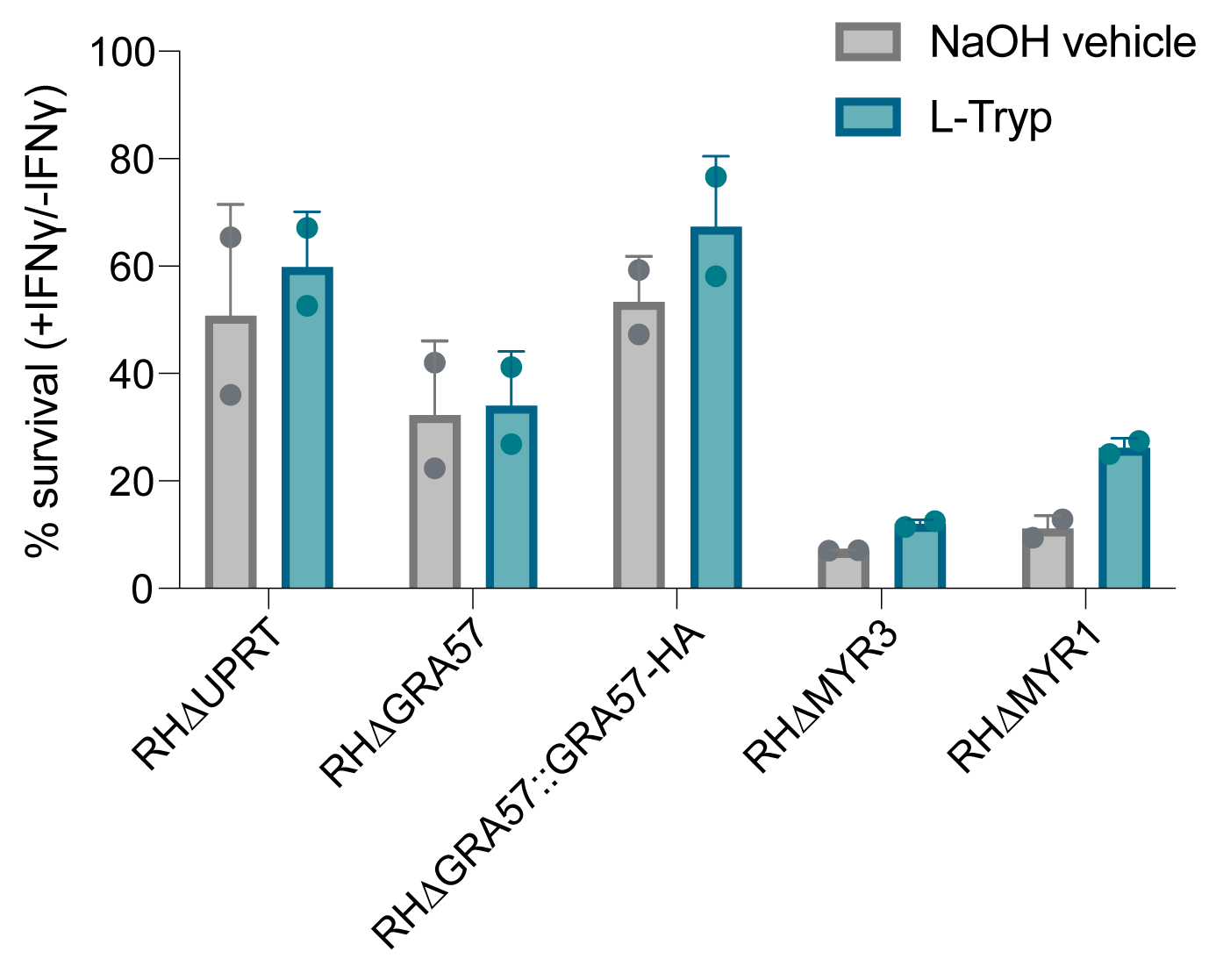

Supplement: S5 Fig — HFF IFNγ restriction assays as in Fig 2, with the addition of 1 mM L-tryptophan to treated conditions simultaneous with IFNγ pre-stimulation. Untreated controls had 0.1 N NaOH added as a vehicle control. Host cells were infected in technical triplicate with the indicated parasite strains for 24 h at an MOI of 0.3, and then imaged live on a Cytation 5 plate reader. Total mCherry signal area per well was measured to determine parasite growth in IFNγ stimulated relative to unstimulated cells. Data displayed as mean survival + standard deviation from 2 biological replicates. Source data can be found in S3 Data. (TIF) [file pbio.3002202.s005.tif]

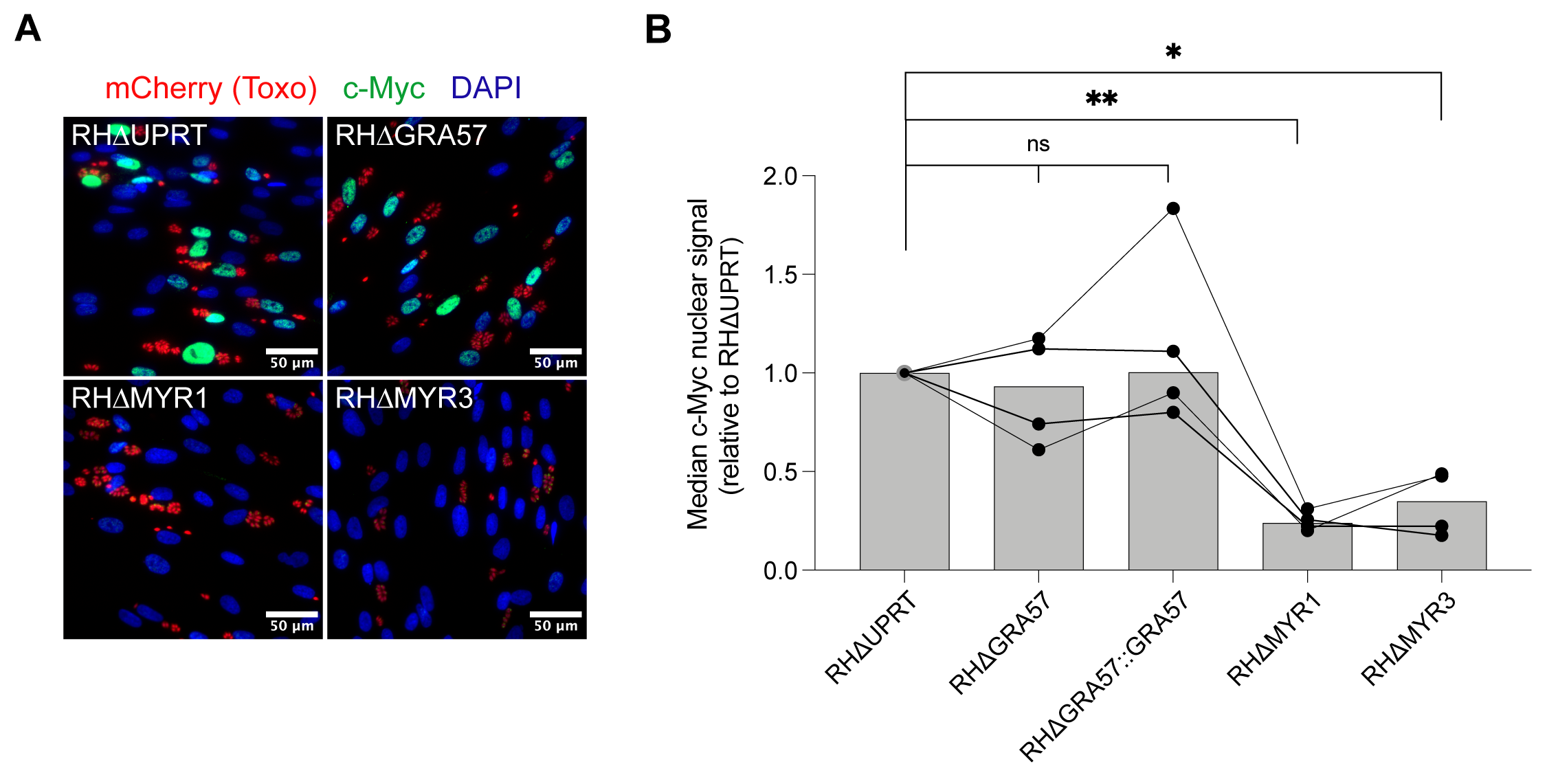

Supplement: S6 Fig — (A) Representative IFA images. HFFs were infected for 24 h prior to fixation and staining for host c-Myc. (B) Quantification of nuclear translocation of host c-Myc. Median nuclear c-Myc signal in infected cells was measured in FIJI, with the median background c-Myc signal subtracted for each image. Median nuclear c-Myc fluorescence intensity for each strain was normalised to that of RHΔUPRT in each biological replicate. Data is shown as median with individual biological replicates overlayed. p-values were calculated by one-way analysis of variance (ANOVA) with Tukey’s multiple comparison test. *, p < 0.05; **, p < 0.01; ns, not significant. Source data can be found in S6 Data. (TIF) [file pbio.3002202.s006.tif]

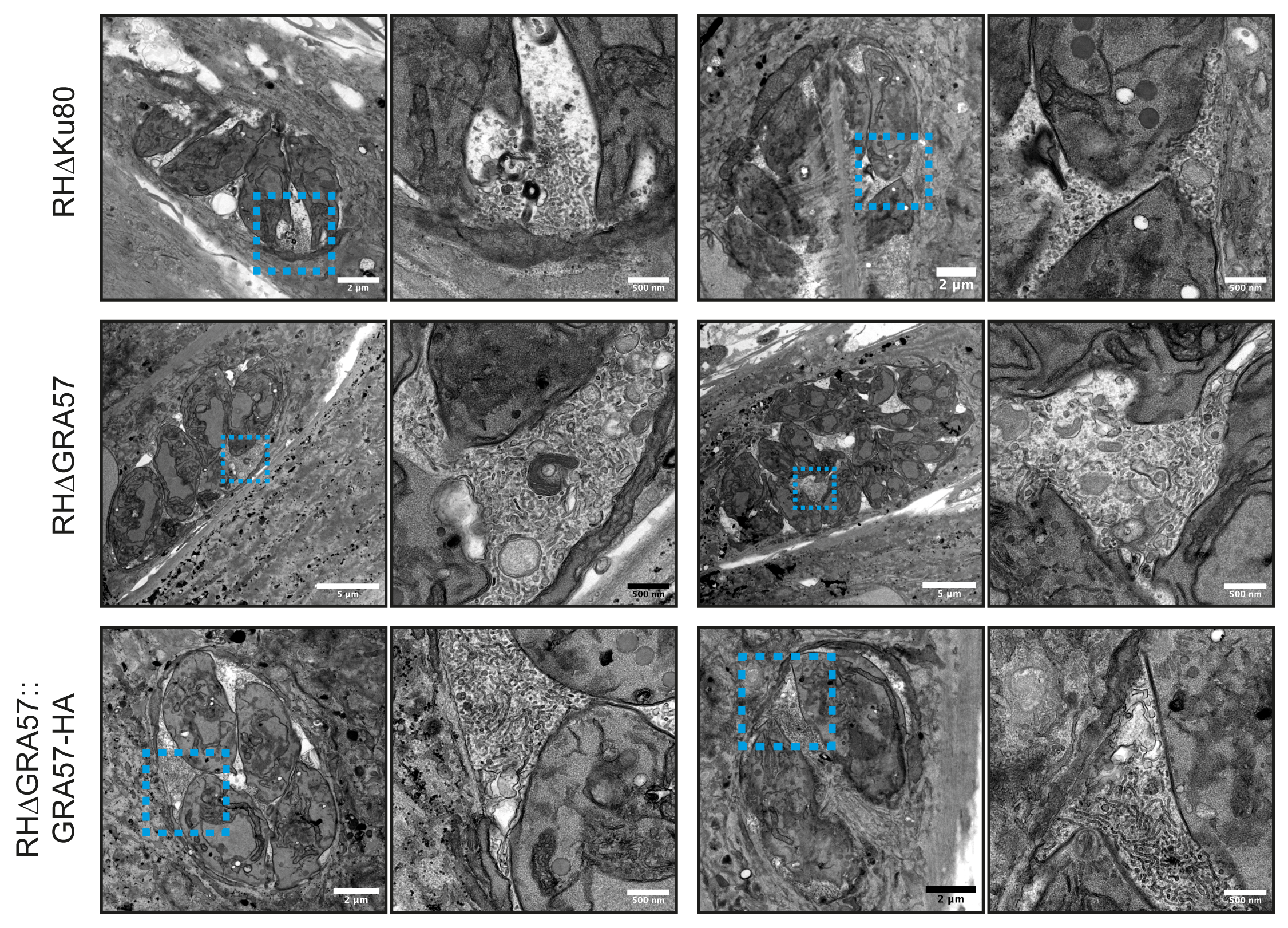

Supplement: S7 Fig — HFFs monolayers were infected with the indicated strains for 24 h prior to fixation and preparation for transmission electron microscopy (TEM). (TIF) [file pbio.3002202.s007.tif]

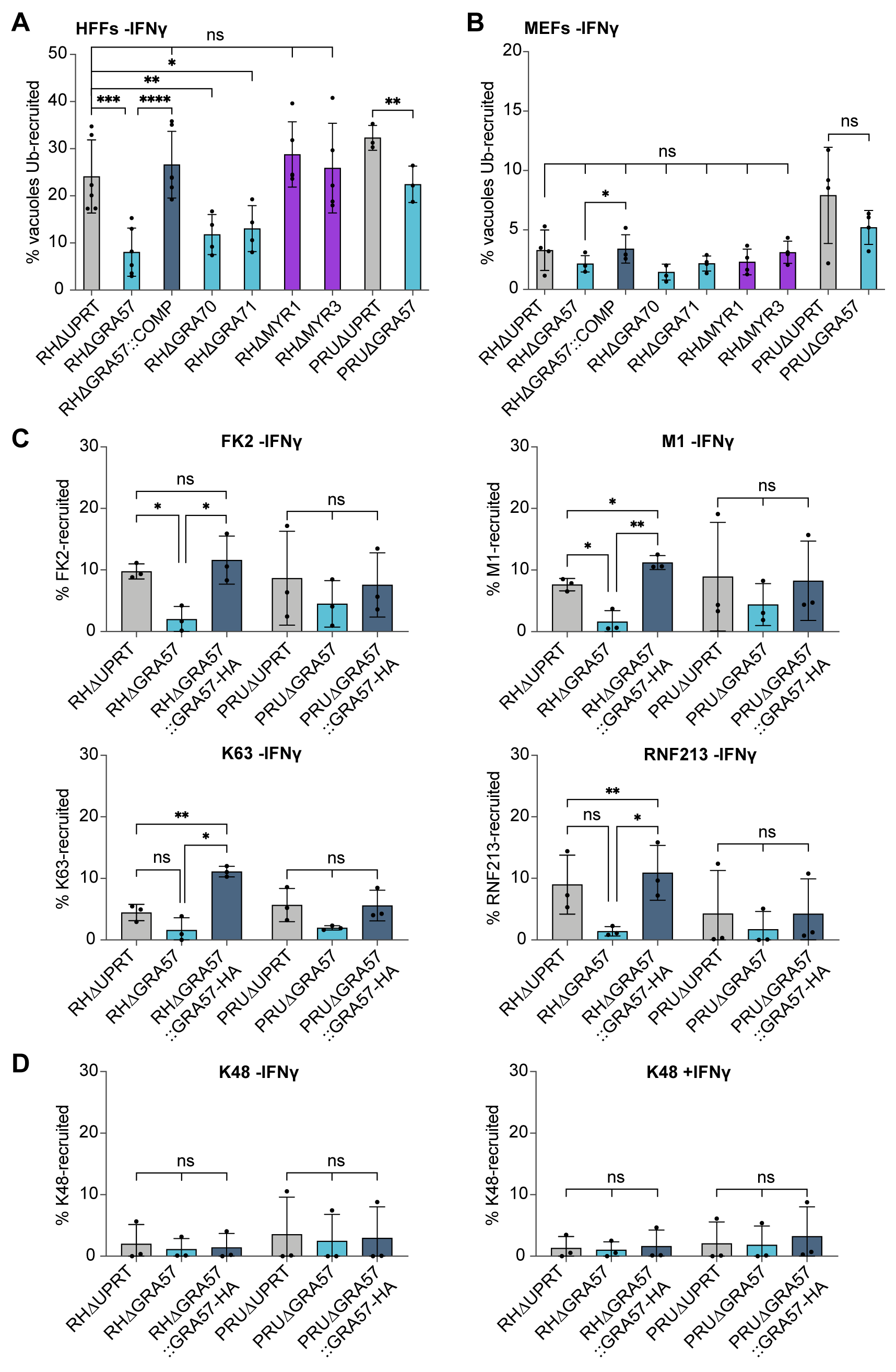

Supplement: S8 Fig — (A, B) Recruitment of total host ubiquitin (FK2) to Toxoplasma vacuoles in (A) HFFs and (B) MEFs. Related to Fig 5A and 5B, data shows ubiquitin recruitment levels in unstimulated cells. Recruitment of ubiquitin was automatically counted using high-content imaging and analysis. p-values were calculated by paired two-sided t test. (C) Recruitment of total ubiquitin (FK2), K63-linked ubiquitin, linear ubiquitin (M1), and the E3 ligase RNF213 to Toxoplasma vacuoles in unstimulated HFFs. Related to Fig 5D, data shows specific ubiquitin linkage recruitment levels in unstimulated HFFs. Recruitment was automatically quantified for FK2, M1, and RNF213. K63 recruitment was manually scored, with minimum 100 vacuoles scored per condition. (D) Recruitment of K48-linked ubiquitin to Toxoplasma vacuoles in HFFs. Host cells were infected and stained for K48-linked ubiquitin as described in Fig 5, with automatic quantification of recruitment. p-values were calculated by paired two-sided t test. *, p < 0.05; **, p < 0.01; ***, p < 0.001; ****, p < 0.0001; ns, not significant. Source data can be found in S9 Data. (TIF) [file pbio.3002202.s008.tif]

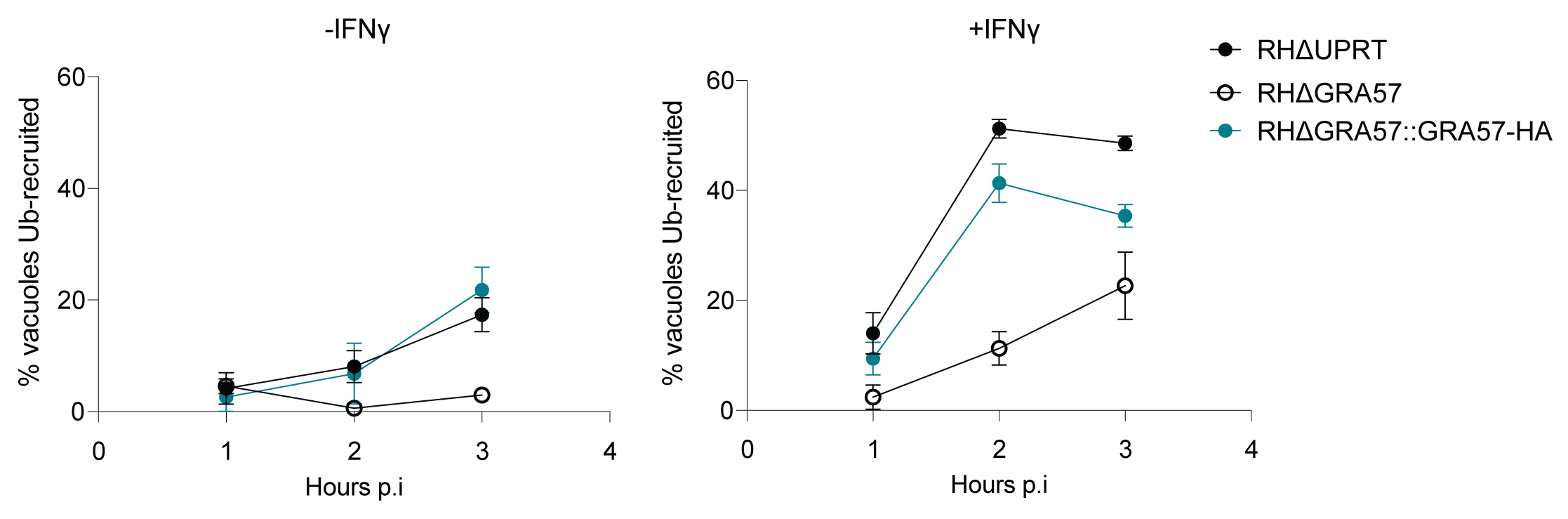

Supplement: S9 Fig — HFFs were infected as in Fig 5, and then fixed at the indicated time points post-infection. Data shown as mean of technical triplicate ± standard deviation. Source data can be found in S9 Data. (TIF) [file pbio.3002202.s009.tif]

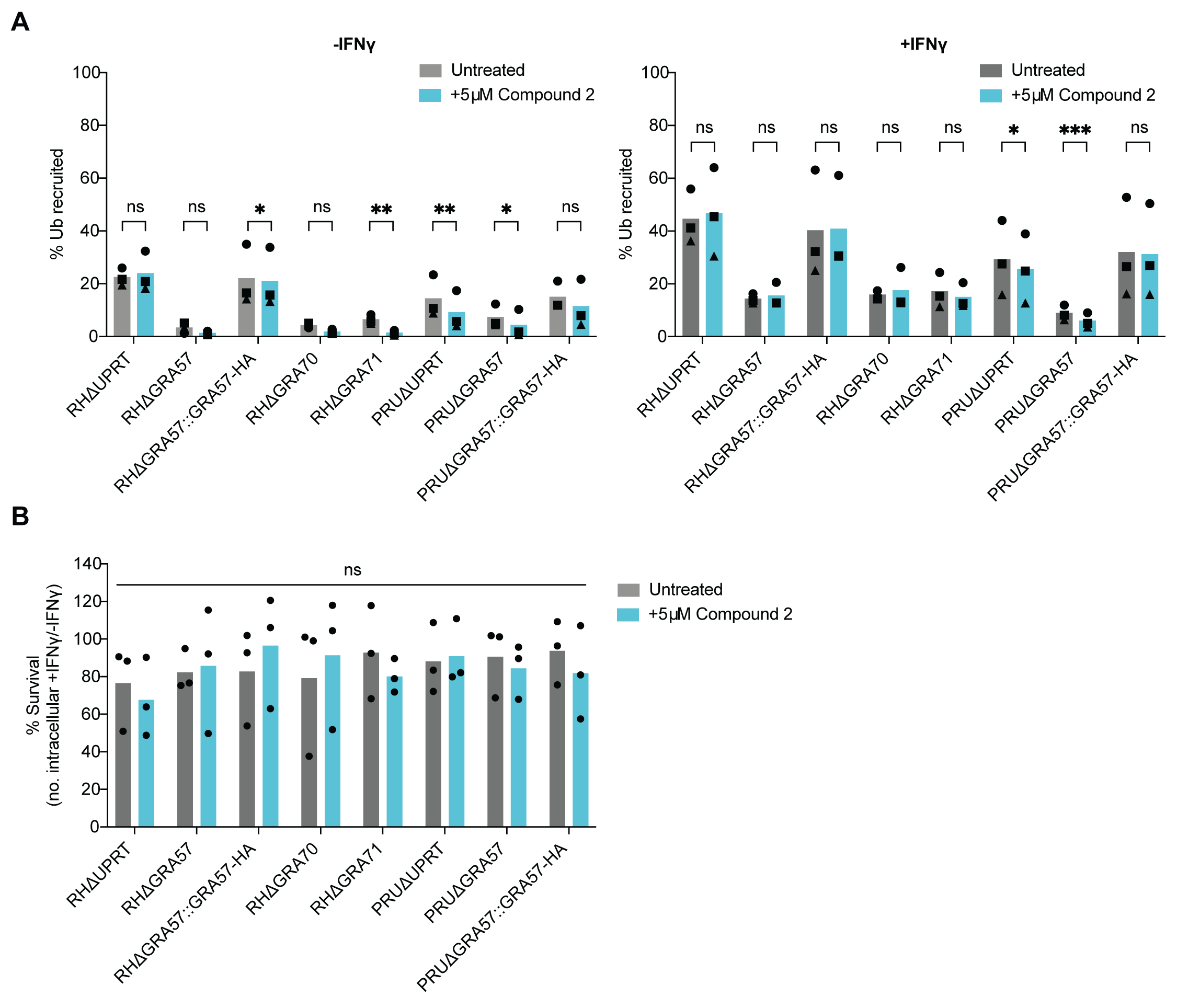

Supplement: S10 Fig — (A) Recruitment of total ubiquitin to Toxoplasma vacuoles at 3 h post-infection, with the addition of 5 μM Compound 2 at 30 min post-infection to inhibit parasite egress. HFFs were pre-stimulated with 100 U/ml IFNγ for 24 h, infected with indicated lines for 30 min prior to addition of Compound 2. HFFs were fixed at 3 h post-infection and stained for total ubiquitin. Recruitment was automatically counted using high-content imaging and analysis. (B) Parasite survival in IFNγ-stimulated HFFs at 3 h post-infection, with the addition of 5 μM Compound 2 at 30 min post-infection to inhibit parasite egress. Parasite numbers were quantified through automated high-content imaging, with survival calculated as the percentage of intracellular parasites in IFNγ-stimulated cells relative to the total in unstimulated cells. p-values were calculated by paired two-sided t test. *, p < 0.05; **, p < 0.01; ***, p < 0.001; ****, p < 0.0001; ns, not significant. Source data can be found in S10 Data. (TIF) [file pbio.3002202.s010.tif]
