# Supplementary material for: A heterotrimeric complex of Toxoplasma proteins promotes parasite survival in interferon gamma-stimulated human cells
Source: PLoS Biol. 2023 Jul 17;21(7):e3002202. doi: 10.1371/journal.pbio.3002202 (PMC10373997; doi:10.1371/journal.pbio.3002202)

S1_Raw_Images Original images for blots and gels

Western blot images used for Fig 2B

Blot was cut in half and top half probed with anti-HA and bottom half with anti-Toxo.

A) Inverted colorimetric image showing prestained molecular weight marker for top half of blot.

B) Chemiluminescent signal for anti-HA-HRP.

C) Merge of images in A and B.

D) Inverted colorimetric image showing prestained molecular weight marker for bottom half of blot.

E) Chemiluminescent signal for rabbit-anti-toxo detected with anti-rabbit-HRP.

F) Merge of images in D and E.

Lane numbers; 1, RHΔKu80, 2, RHΔGRA57, RHΔGRA57::GRA57-HA.

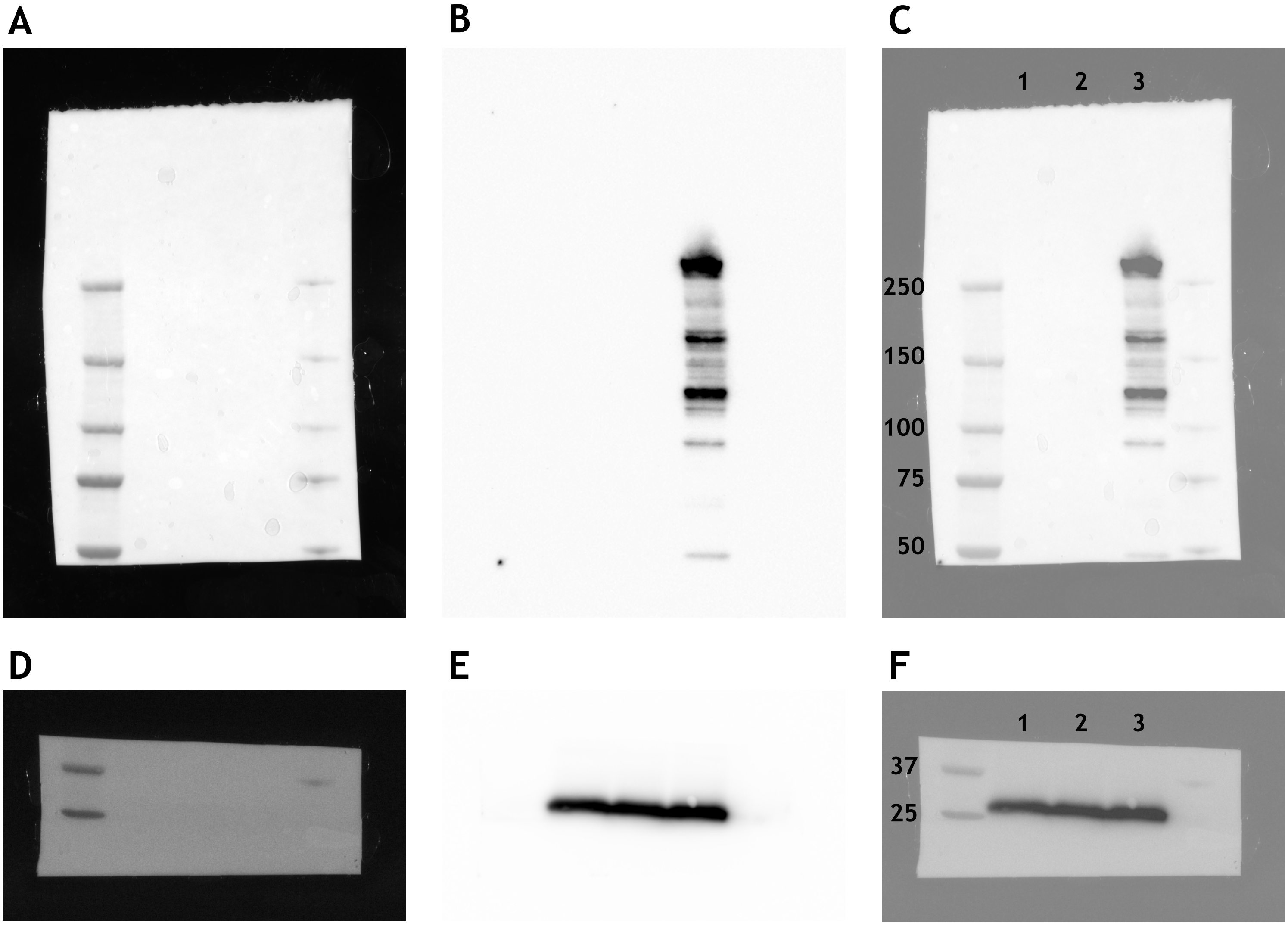

Western blot images used for Fig 3D

Blot was cut in half and top half probed with anti-V5, then anti-HA, and bottom half with anti-Toxo.

A) Inverted colorimetric image showing prestained molecular weight marker for top half of blot.

B) Chemiluminescent signal for rabbit-anti-V5 detected with anti-Rabbit-HRP.

C) Merge of images in A and B.

D) Inverted colorimetric image showing prestained molecular weight marker for top half of blot.

E) Chemiluminescent signal for anti-HA-HRP.

F) Merge of images in D and E.

G) Inverted colorimetric image showing prestained molecular weight marker for bottom half of blot.

H) Chemiluminescent signal for rabbit-anti-toxo detected with anti-rabbit-HRP.

I) Merge of images in D and E.

Lane numbers: 1-RHΔKu80, 2-RHGRA57-HA, 3-empty, 4- RHΔKu80, 5-RHGRA70-V5, 6- unrelated to this report.

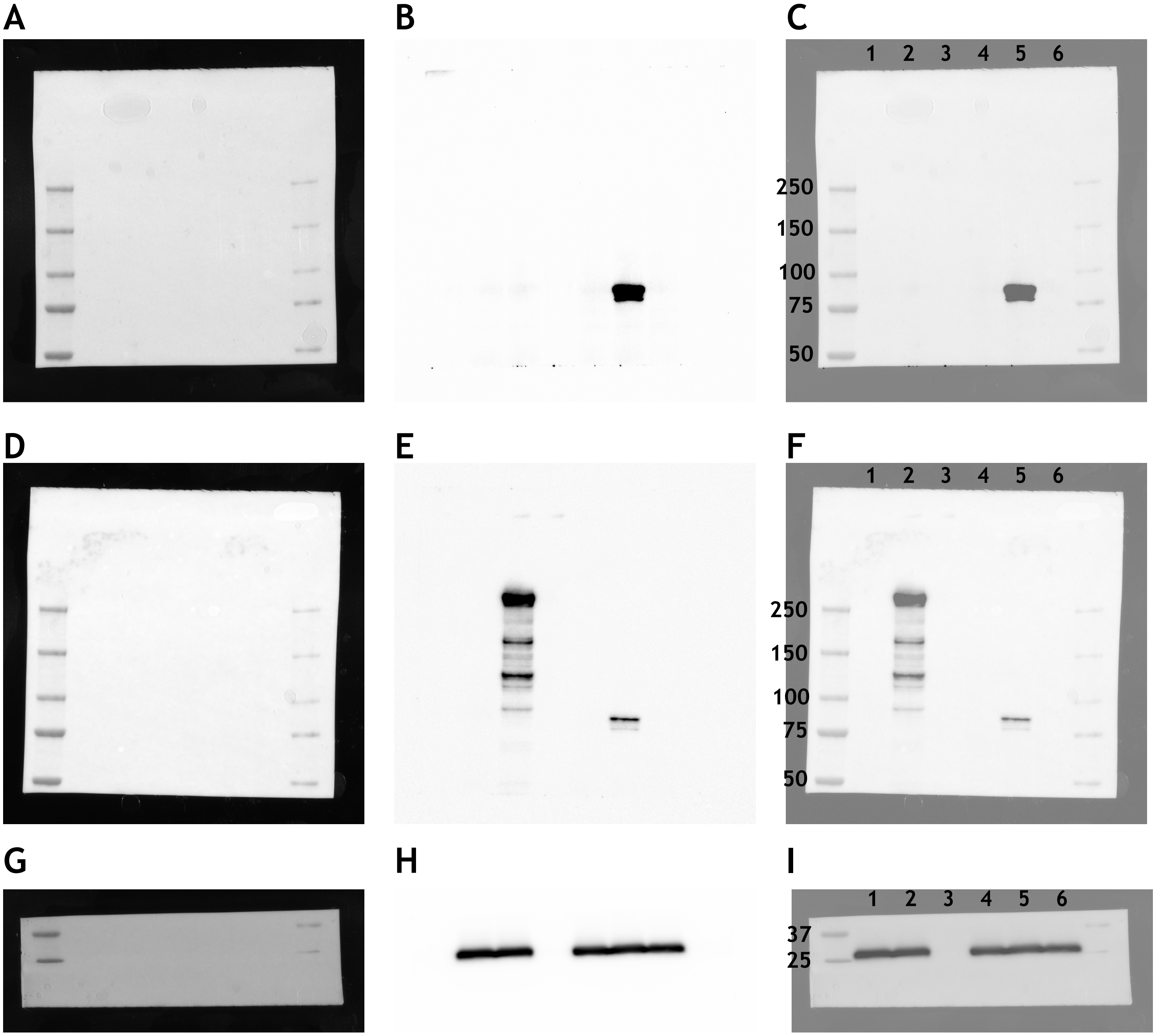

DNA gels used in Fig S1A.
Box denotes regions used in figure, unboxed lanes are unrelated to this figure.

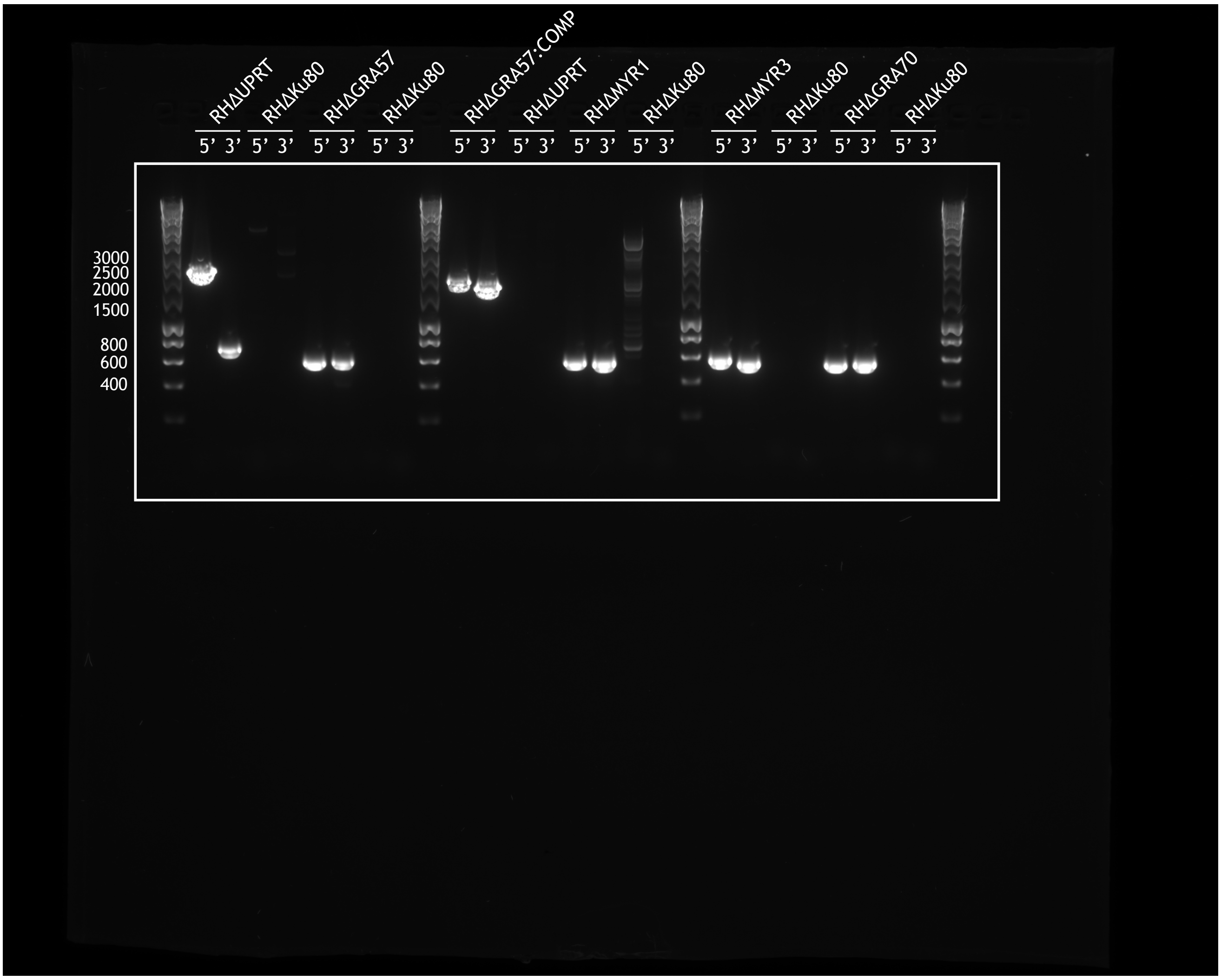

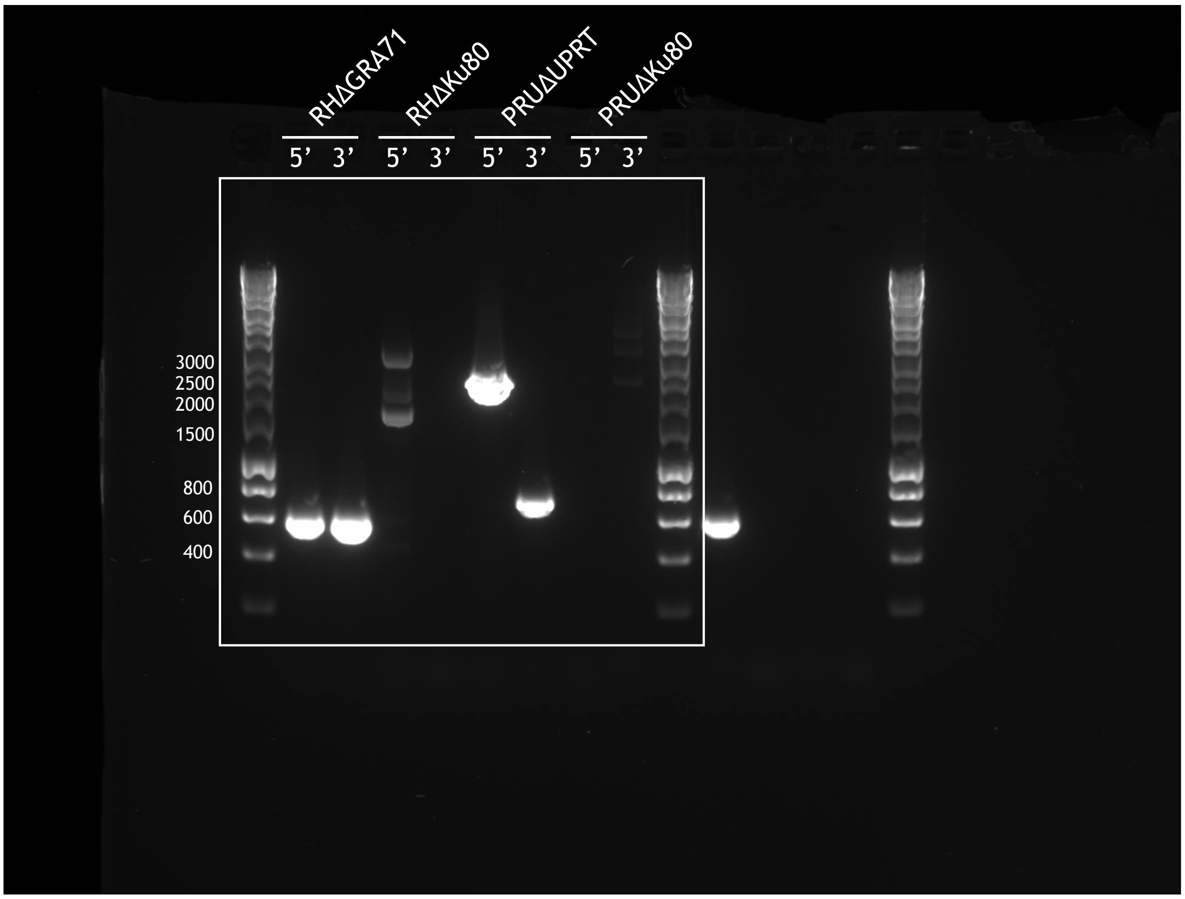

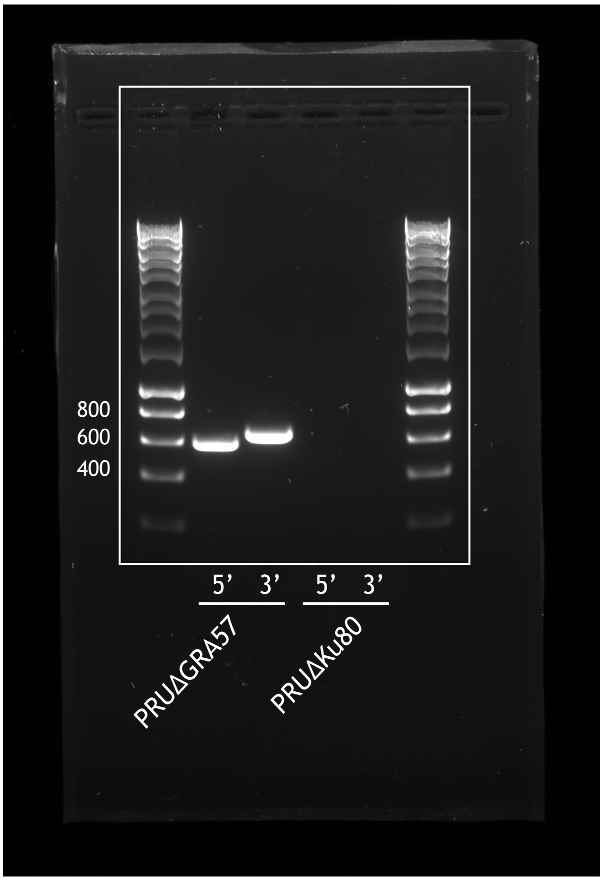
800
600
400
5'
3'
5'
3'
PRUΔGRA57
PRUΔKu80

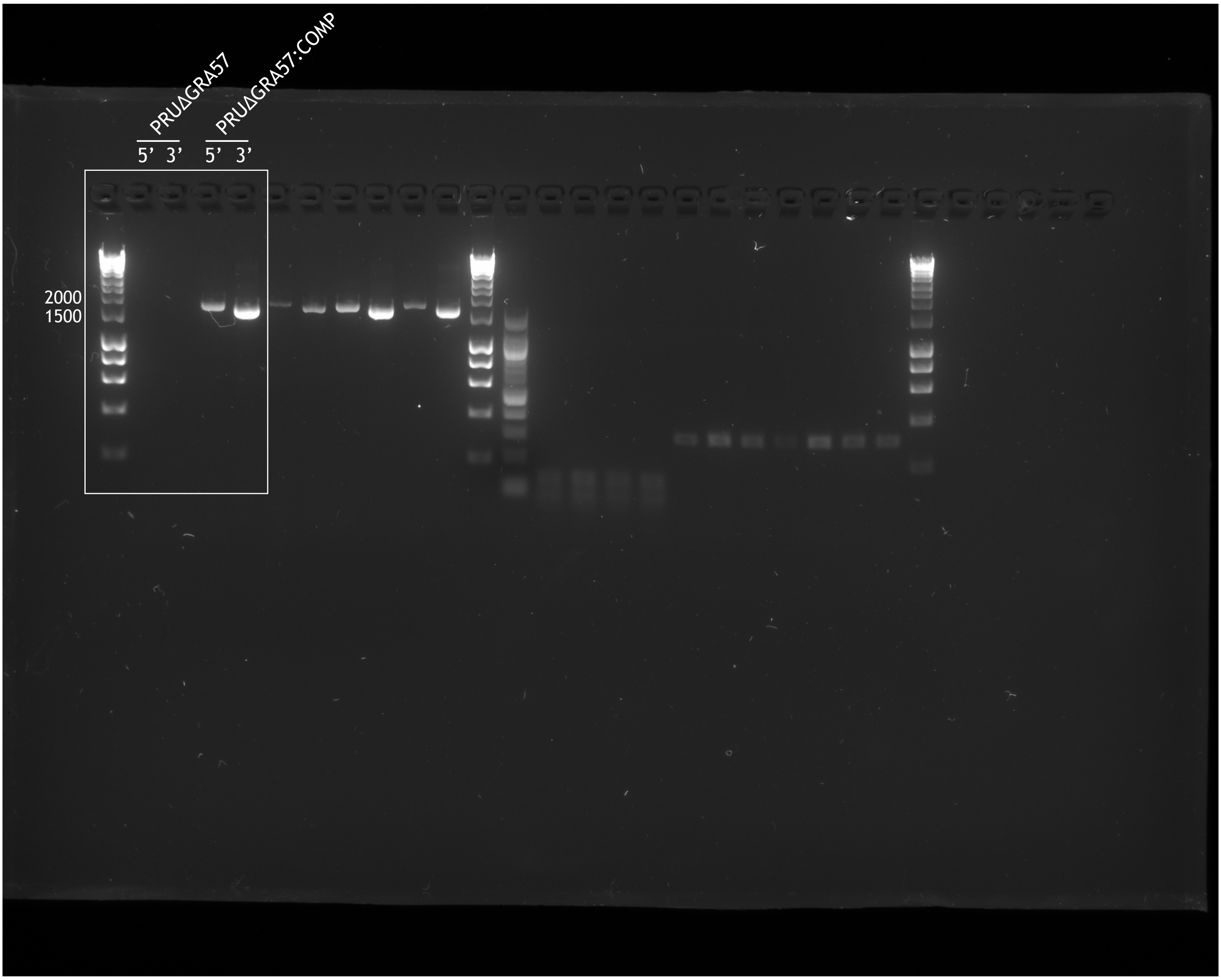
PRUΔGRA57
PRUΔGRA57:COMP
5'
3'
5'
3'
2000
1500

DNA gels used in Fig S1B.

Box denotes regions used in figure, unboxed lanes are unrelated to this figure. X indicates lanes unrelated to this report.

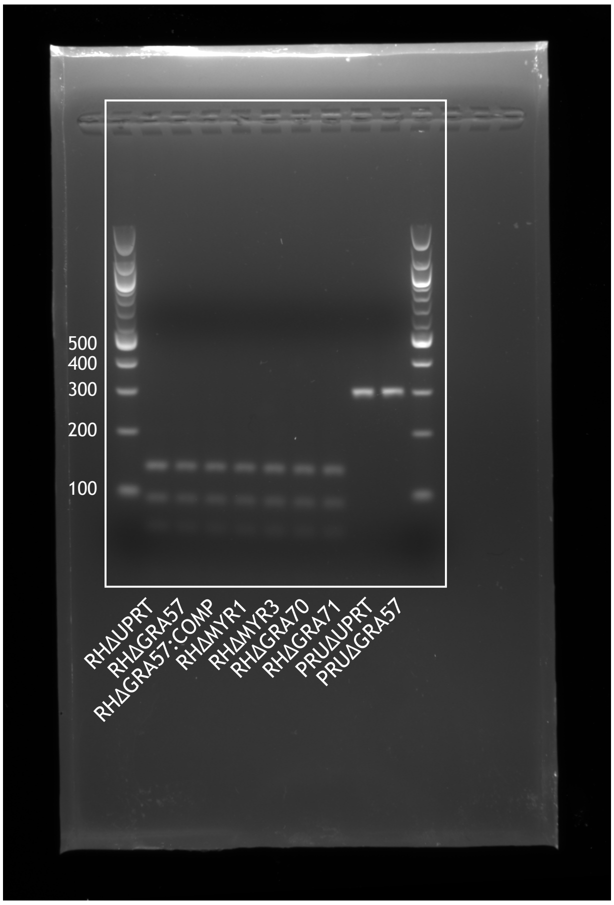

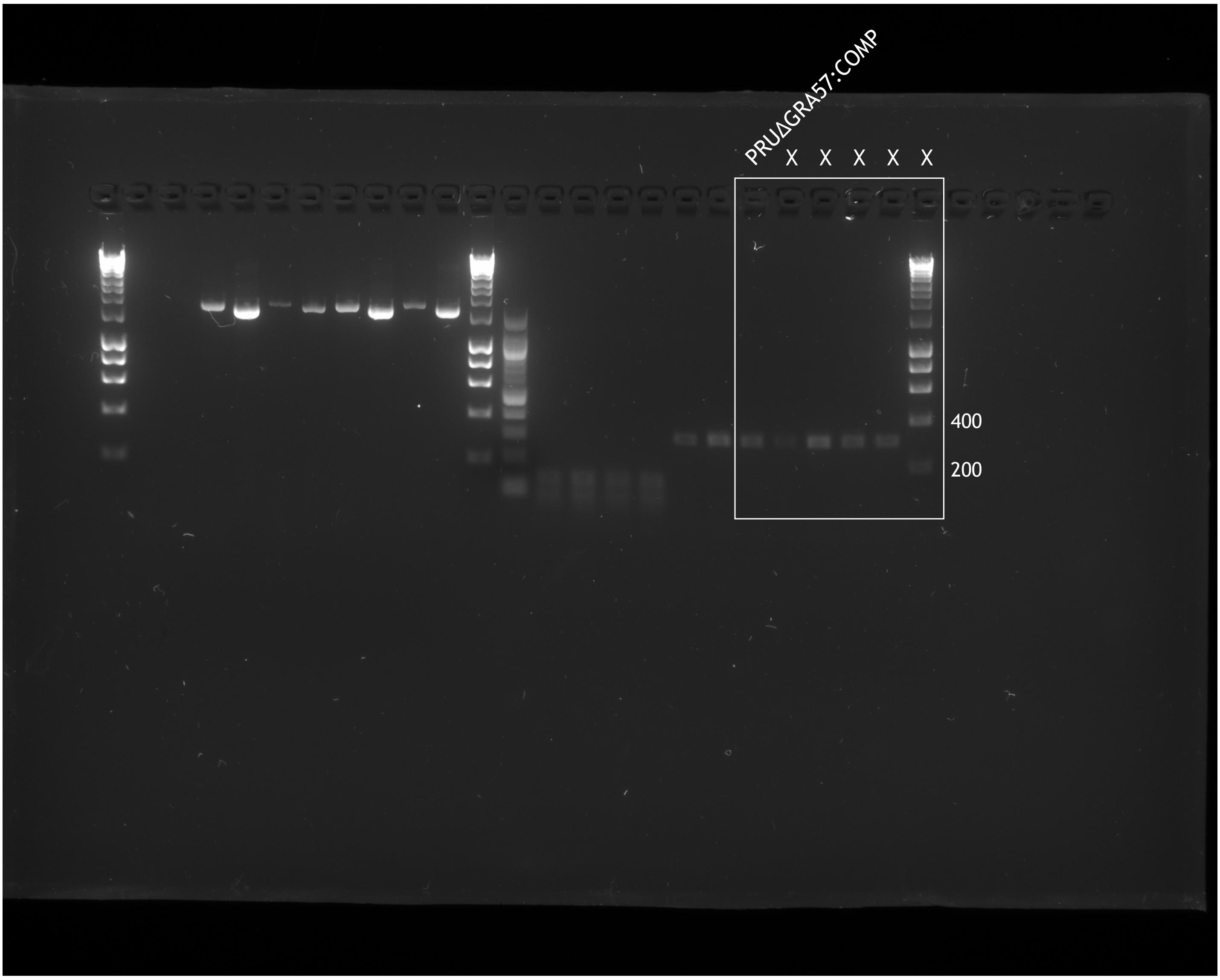

DNA gels used in Figure S2.
Box denotes regions used in figure, unboxed lanes are unrelated to this figure.

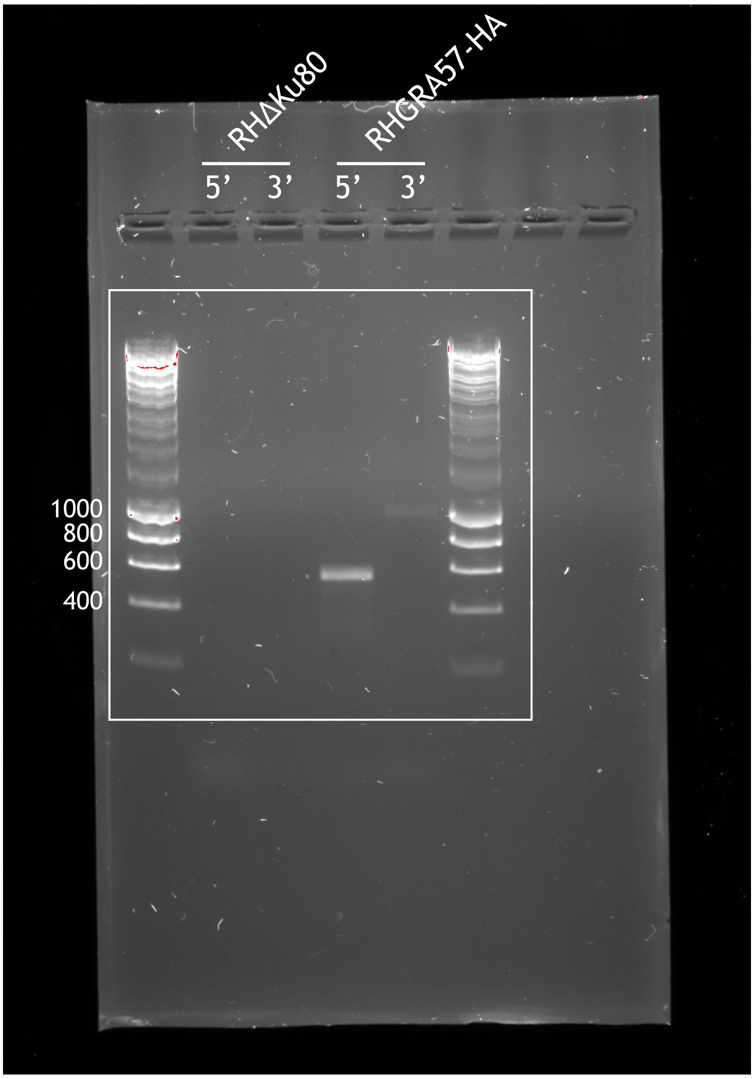

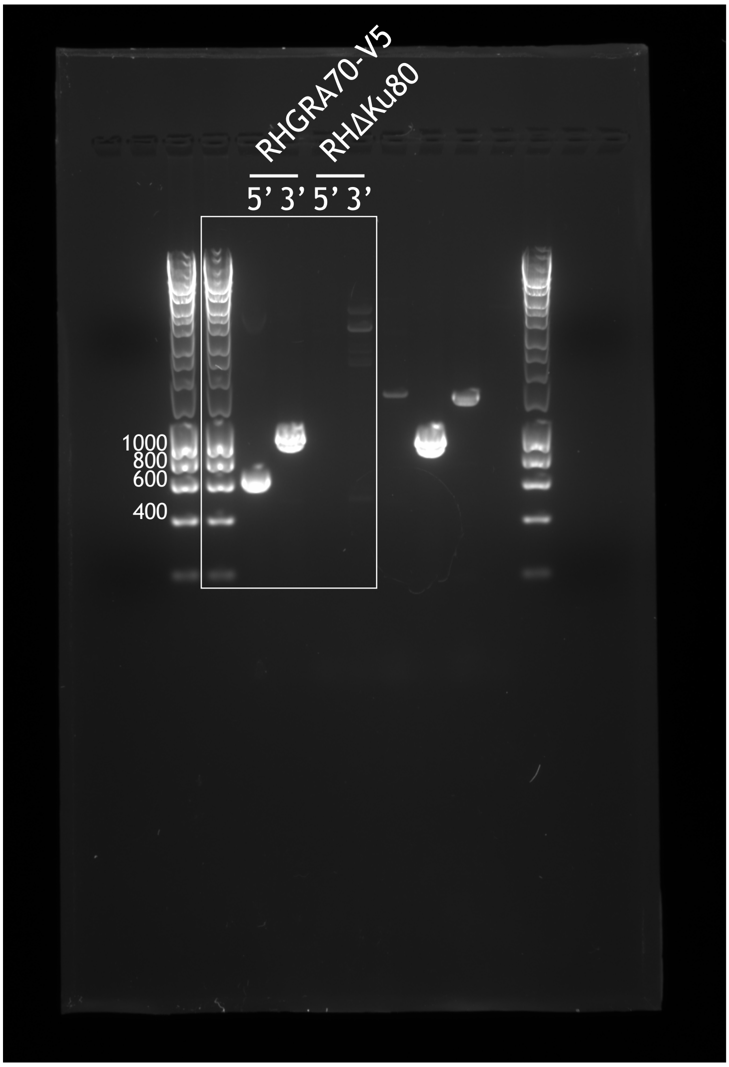

Supplement: S1 Raw Images — (PDF) [file pbio.3002202.s011.pdf]
