# Supplementary material for: A heterotrimeric complex of Toxoplasma proteins promotes parasite survival in interferon gamma-stimulated human cells
Source: PLoS Biol. 2023 Jul 17;21(7):e3002202. doi: 10.1371/journal.pbio.3002202 (PMC10373997; doi:10.1371/journal.pbio.3002202)
Supplement: S12 Data — (DOCX) [file pbio.3002202.s023.docx]

**Opera Phenix acquisition parameters- HFF and MEF ubiquitin recruitment**

|  | Parameter using 40X Water, NA 1.1 | Ex (nm) / Em (nm) | | | |
| --- | --- | --- | --- | --- | --- |
|  |  | 405 / 450 | 488 / 525 | 568 /600 | 647 /690 |
| Extracellular parasite CH: 647 | Time |  | 40 ~ 100 ms | 100 ms | 20 ~ 40 ms |
|  | Power |  | 100 % | 100 % | 100 % |
| Extracellular parasite CH: 405 | Time | 100 ms | 60 ms | 40 ms |  |
|  | Power | 100 % | 100 % | 100 % |  |

Z-stack: 5 focal planes from -1 to 1 µm with a step size of 0.5 µm

**Harmony v5.0 Analysis sequence to determine ubiquitin-recruited Toxoplasma within HFFs and MEFs**

| Step | Building block | Input / Method / Output |
| --- | --- | --- |
| 1 | Input Image | Channel group: 1, Sequences: ALL, Flatfield Correction: None, Brightfield Correction, Stack Processing: Maximum Projection |
| 2 | Find Image Region | Channel: Alexa 568, ROI: None / Method: Whole Image Region / Output Population: Whole Image, Output Region: Whole Image Region |
| 3 | Calculate Intensity Properties | Channel: Alexa 488, Population: Whole Image Region: Whole Image Region / Method: Standard, Mean, Median / Property Prefix: Intensity Whole Image, Region Alexa 488 |
| 4 | Find Spots | Channel: Alexa 568, ROI: None / Method: B, Detection Sensitivity: 0.5, Splitting Sensitivity: 0.5, Calculate Spot Properties / Output Population: mCh+ vacuole candidate |
| 5 | Select Population | Population: mCh+ vacuole candidate / Method: Filter by Property  Spot Area [px²]: > 50 / Output Population: mCh+ vacuole |
| 6 | Select Region | Population: mCh+ vacuole, Region: Spot / Method: Resize Region  [μm/px], Outer Border: -1.5 μm, Restrictive Population: None, Inner Border: INF μm / Output Region: Vacuole region |
| 7 | Select Region (2) | Population: mCh+ vacuole, Region: Spot / Method: Resize Region [μm/px],  Outer Border: -3 μm, Restrictive Population: None, Inner Border: -2 μm / Output Region: Cell region |
| 8 | Calculate Intensity  Properties (2) | Channel: Alexa 488, Population: mCh+ vacuole, Region: Vacuole region / Method: Standard, Mean / Property Prefix: Intensity Vacuole region Alexa 488 |
| 9 | Calculate Intensity  Properties (3) | Channel: Alexa 488, Population: mCh+ vacuole, Region: Cell region / Method: Standard, Mean / Property Prefix: Intensity Cell region Alexa 488 |
| 10 | Calculate Properties | Population: mCh+ vacuole / Method: By Formula, Formula: A/B, Variable A: Intensity Vacuole region Alexa 488 Mean, Variable B: Intensity Cell region Alexa 488 Mean / Output Property: Ratio  488 vacuole / cell |
| 11 | Calculate Intensity  Properties (4) | Channel: Alexa 647 (or DAPI), Population: mCh+ vacuole, Region: Spot / Method: Standard, Mean / Property Prefix: Intensity Spot Alexa 647 (or DAPI) |
| 12 | Select Population (2) | Population: mCh+ vacuole / Method: Filter by Property, Intensity Spot Alexa 647 Mean: > 4500 ~ 6500 (or DAPI Mean: > 700), Intensity Spot Alexa 647 CV [%]: <50 (or DAPI CV [%]: <25), Boolean Operations: F1 and F2 / Output Population: Extracellular Toxo |
| 13 | Select Population (3) | Population: mCh+ vacuole / Method: Filter by Property, Extracellular Toxo: ==0 / Output Population: intracellular vacuole |
| 14 | Select Population (4) | Population: intracellular vacuole / Method: Filter by Property, Ratio 488 vacuole / cell: > 1.3 ~ 1.8 (FK2), > 2 ~ 2.5 (M1), > 1.5 ~ 1.8 (K48), > 2.5 (RNF213) Intensity Vacuole region Alexa 488 Mean: > 600 ~ 1000 (FK2), > 2000 (M1), > 480 (K48), > 2000 (RNF213), Boolean Operations: F1 and F2 / Output Population: intracellular 488-recruited vacuole |
| 15 | Define Results | Method: List of Outputs / Population: intracellular vacuole, Number of Objects / Population: mCh+ vacuole, Number of Objects / Population: intracellular 488-recruited vacuole, Number of Objects, Method: Formula Output, Formula: a/b*100, Population Type: Objects, Variable a: intracellular 488-recruited vacuole - Number of Objects, Variable b: intracellular vacuole - Number of Objects, Output Name: % recruitment / Method: Formula Output, Formula: a/b*100, Population Type: Objects, Variable a: intracellular vacuole - Number of Objects, Variable b: mCh+ vacuole - Number of Objects, Output Name: % of intracellular vacuole in total vacuole |
