# Supplementary material for: A heterotrimeric complex of Toxoplasma proteins promotes parasite survival in interferon gamma-stimulated human cells
Source: PLoS Biol. 2023 Jul 17;21(7):e3002202. doi: 10.1371/journal.pbio.3002202 (PMC10373997; doi:10.1371/journal.pbio.3002202)
Supplement: S13 Data — (DOCX) [file pbio.3002202.s024.docx]

**Primary antibodies used for immunofluorescence assays**

| **Target** | **Species** | **Dilution** | **Source** |
| --- | --- | --- | --- |
| HA | Rat | 1:500 | Roche, 11867423001 |
| V5 | Mouse | 1:200 | Abcam, ab27671 |
| V5 | Rabbit | 1:200 | Abcam, MA5-32053 |
| GRA2 | Mouse | 1:1000 | BioTem, BIO.018.5 |
| GRA3 | Rabbit | 1:1000 | Gift from Jean François Dubremetz |

**Secondary antibodies used for immunofluorescence assays**

| **Target** | **Fluorophore** | **Species** | **Dilution** | **Source** |
| --- | --- | --- | --- | --- |
| Rat | Alexa 488 | Donkey | 1:2000 | ThermoFisher, A21208 |
| Mouse | Alexa 488 Plus | Donkey | 1:2000 | ThermoFisher, A32766 |
| Rabbit | Alexa 488 Plus | Donkey | 1:2000 | ThermoFisher, A32790 |
| Mouse | Alexa 647 | Goat | 1:500 | ThermoFisher, A21235 |
| Rabbit | Alexa 647 | Goat | 1:500 | ThermoFisher, A21244 |
