# Supplementary material for: A heterotrimeric complex of Toxoplasma proteins promotes parasite survival in interferon gamma-stimulated human cells
Source: PLoS Biol. 2023 Jul 17;21(7):e3002202. doi: 10.1371/journal.pbio.3002202 (PMC10373997; doi:10.1371/journal.pbio.3002202)
Supplement: Attachment — Submitted filename: 132311_1_rebuttal_2642108_rsjgyp.docx [file pbio.3002202.s025.docx]

**Manuscript number:** RC-2022-01786R

**Corresponding author(s):** Moritz, Treeck

*[Please use this template only if the submitted manuscript should be considered by the affiliate journal as a full revision in response to the points raised by the reviewers.*

*If you wish to submit a preliminary revision with a revision plan, please use our "*[*Revision Plan*](https://docs.google.com/document/u/0/d/1HHp62Tfeb44nd3Gu_3ChB0b8gmMdEog7GjZcZFwHSiQ/edit)*" template.* ***It is important to use the appropriate template to clearly inform the editors of your intentions.****]*

## 1. General Statements [optional]

We would truly like to thank all 3 reviewers for insightful, helpful and thus constructive comments.

## 2. Point-by-point description of the revisions

*This section is mandatory.* *Please insert a point-by-point reply describing the revisions that were already carried out and included in the transferred manuscript.*

NB: Reviews are quoted in italics; responses are indented

Reviewer #1

**Summary**

*In this manuscript, Lockyer et al. provide novel insights into the mechanism by which Toxoplasma gondii avoids parasite restriction in IFNγ-activated human cells. To identify potentially secreted proteins supporting parasite survival in IFNγ-activated human foreskin fibroblasts (HFF), the authors designed a CRISPR screen of Toxoplasma secretome candidates based on hyperLOPIT protein localization data. By this approach, they identified novel secreted proteins supporting parasite growth in IFNγ-activated cells. Among the gene identified, they found MYR3 a known component of the putative translocon in charge of protein export through the parasitophorous vacuole membrane. Therefore, the authors focused their investigations on GRA57, a dense granule protein of unknown function, which affects parasite survival to a lesser extent than the MYR component. The resistance phenotype conferred by GRA57 was confirmed by fluorescence microscopy. Importantly, the authors provide evidence that the protective function of GRA57 is not as well conserved in murine cells of the same type (MEF) as in HFF. To further explore the mechanism by which GRA57 protect the parasites in IFNγ-activated cells, the authors searched for protein partners by biochemistry. By immunoprecipitation and tandem mass spectrometry, they identified two other putative dense granule proteins, GRA70 and GRA71, which co-purified with GRA57-HA tagged protein. Noteworthy, both proteins were also found in the CRISPR screens with significant score conferring resistance. High-content imaging analysis confirmed the protective effect conferred by GRA57, GRA70, and GRA71 individually at similar levels. After ruling out an effect of tryptophan deprivation in parasite clearance, or a role of GRA57 in protein export normally mediated by the MYR translocon, and a role on host cell gene expression by RNA-Seq, the authors investigated the ubiquitination of the parasitophorous vacuole membrane, a marker previously thought to initiate parasite clearance. A reduction in ubiquitin labeling around the vacuole of mutant parasites is observed, which is quite surprising given the correlated increase in parasite clearance. The authors concluded that ubiquitin recruitment may not be directly linked to the parasite clearance mechanism.*

**Major comments**

- *Figure 2C. In this figure, the restriction effect of IFNγ is about 60% (or 40% survival) for RHdeltaUPRT parasites grown in HFFs, which is quite different from the 85% mentioned earlier in the results section. How was actually done the first assay? Settings with 60% restriction sounds reasonable and indicates that a substantial fraction of the parasite population evades the restrictive effect of IFNγ, which provides a clear rationale for the main objective of this study, namely the identification of effectors supporting parasite development in human cells in the presence of IFNγ.*

This discrepancy in restriction likely arises from the differences in the parasites used in these assays and the measurements of restriction. The 85%/90% restriction initially mentioned is from the pooled CRISPR screens using the effector knockout pool. This restriction level was assessed by counting of parasites retrieved following infection of IFNγ-stimulated HFFs. The 60% restriction of wildtype parasites seen in Figure 2 is a separate assay. This percentage was calculated by measuring total mCherry fluorescence area within infected HFFs. We expect the restriction of the pooled CRISPR population to be higher than in restriction assays performed with either wild type parasites or single genetic knockouts. We included the 85%/90% numbers to highlight that the HFFs were highly restrictive in the screen, but we have now removed references to these numbers in the results section to avoid confusion with later results that use more accurate measures of survival. We refer to this restriction level instead in the discussion section.

*Optional comment: GRA70 and GRA71 were both copurified with GRA57, but what about GRA71 expression and localization? Is there a reason why this protein partner has not been studied further just like GRA70?*

Tagging of GRA71 was attempted but was not successful in a first attempt. We have not re-attempted this tagging as Krishnamurthy et al 2023 (PMID: 36916910) recently tagged and localised GRA71, demonstrating it is also an intravacuolar dense granule protein with similar localisation to GRA57 and GRA70- we feel there is minimal value in us repeating this.

*Is there any change in GRA57, GRA70, and GRA71 localization and/or amount when cells were pretreated with IFNγ?*

Thank you for this suggestion, we have now conducted further investigation to address this. We checked the localisation of GRA57-HA and GRA70-V5 in IFNγ-stimulated HFFs and found no change to their localisation. This data has been added in Supplementary Figure S4 in our revised manuscript. Alignment of our RNA-Seq data to the *Toxoplasma* genome, now included as Supplementary Data 4, also shows there is no significant up or downregulation in expression of any of the three proteins when HFFs are pretreated with IFNγ.

*Do they still form a complex in the absence of IFNγ?*

We did not investigate this in this manuscript, however in Krishnamurthy et al 2023 (PMID: 36916910) CoIPs using GRA57 and GRA70 in the absence of IFNγ also identified these three proteins as interaction partners, so formation of the complex is likely IFNγ-independent.

*In the absence of GRA70 or GRA71 is GRA57 expression and/or localization affected?*

We did not investigate this possibility in this manuscript, however doing so would require the generation of epitope tagged lines in knockout backgrounds. We believe this represents a significant body of work and would therefore be suitable for a future study focused on the further characterisation of this complex. The RNA-Seq data shows that GRA70 and GRA71 expression levels are not significantly different in the RH∆GRA57 strain (Supplementary Data 4) which we have now included as a statement in the results section.

- *Page 13, result section. To determine whether GRA57 has any direct or indirect effect on host cell gene expression, the authors performed RNA-Seq analysis of HFF cells pretreated or not with IFNγ. First, as for proteomic data, were the data deposited on GEO or another repository database?*

*Second, were any effect detected on parasite gene expression? Reads alignment could be done using the T. gondii reference genome to determine whether IFNg or gra57 KO has any effect on parasite genes. Possibly, other secreted proteins not necessarily expressed at the tachyzoite stage and therefore not captured in the hyperLOPIT protein analysis are specifically expressed in these conditions.*

We will deposit the RNA-Seq data on GEO prior to final publication. We did perform read alignment using the *Toxoplasma* *gondii* reference genome, and we agree it would be useful to include this analysis. We have now provided this data in Supplementary Data 4. Comparison of parasite gene expression between RH∆Ku80 and RH∆GRA57 revealed very few major changes (L2FC <-2 or >2) that were also rescued in the RH∆GRA57::GRA57 line, irrespective of IFNγ stimulation. Of the few genes that were up or downregulated in the RH∆GRA57 parasites, these were all uncharacterised. Collectively this data did not provide any mechanistic insight into the function of GRA57, and we think it unlikely the GRA57 phenotype is related to major changes in host or parasite gene expression. We have amended the manuscript to highlight this.

*Optional comment: RNA-Seq analysis points to a clear induction of GBPs upon IFNγ treatment in HFF. Given the clear function of GBP in parasite clearance, have the authors ever hypothesized that GRA57 could be involved in preventing GBP binding to the PVM?*

We have not tested if GBP recruitment is influenced by GRA57, however GBPs have previously been shown to be dispensable for restriction of *Toxoplasma* growth in HFFs (Niedelman et al 2013, PMID: 24042117) despite being robustly induced by IFNγ stimulation (Kim et al 2007, PMID: 17404298). We have modified the manuscript to highlight this.

**Minor comments**

- *Page 4, introduction, 8th paragraph. Regarding the role of IST, it might be less prone to controversy to state: 'a condition that may only be met in the early stages of infection.'*

We agree and have changed this.

- *Page 4, end of introduction. Changing '... indicating that the three proteins function in a complex'. Changing to '... indicating that the three proteins function in the same pathway.' might be more appropriate for the conclusion.*

We agree and have changed this.

- *Page 4, result section, first paragraph. 'strain specific and independent effectors'. Are the authors talking about strain-specific and non-strain-specific factors?*

Yes- we have changed the text to reflect this.

*- Page 6, result section. 'GRA25, an essential virulence factor in mice'. It is not clear to the reviewer how a virulence factor is essential since both parasite and mouse survival is achieved in the GRA25 mutant. I suggest to replace 'essential' by 'major'.*

We agree and have changed this.

*- Page 7. 'showing that GRA57 resides in the intravacuolar network (IVN) (Figure 2A)'. From the image shown, GRA57 clearly localizes into the PV, but it is hard to tell whether GRA57 is associated with the intravacuolar network. Colocalization assay or electron microscopy would be necessary to draw such conclusions.*

We agree and have changed all references to this localisation as ‘intravacuolar’ instead of specifically the IVN.

*- 'uprt locus'. Lower case letters and italic are generally preferred to designate mutants, whereas upper case letters are generally used for wild type alleles. (Sibley et al., Parasitology Today, 1991. Proposal for a uniform genetic nomenclature in Toxoplasma gondii).*

We agree and have changed this.

*- The authors mentioned in the introduction that ROP1 contributes to T. gondii resistance to IFNγ in murine and human macrophages. However, they did not comment on whether ROP1 was found important in the screen performed here in human HFF cells. It may be useful to reference ROP1 in Figure 1 as GRA15, GRA25, etc.*

ROP1 was not found to be important in the HFF screens (+IFNγ L2FCs in RH: -0.1, PRU: -0.46). As ROP1 was characterised as an IFNγ resistance effector in macrophages, this discrepancy may therefore represent a cell type-specific difference, so we feel it is not relevant to highlight for the purposes of the screens presented here.

*- Figure 2D. The authors compared the restriction effect of IFNγ on parasites grown in HFF and MEF host cells. However, as represented - % + IFNγ/- IFNγ - it cannot be estimated whether the parasites grew similarly in the two host cell types in the absence of IFNγ. Please indicate whether or not the growth was similar in both cell types.*

As these restriction assays were not carried out concurrently and were designed to measure IFNγ survival, we feel it would be inaccurate to compare parasite growth between the two cell types using this data. The focus of these experiments was to investigate the restrictive effect of IFNγ across parasite strains, using the -IFNγ condition to control for differences in growth rate or MOI. Therefore we feel it is appropriate for the focus of our manuscript to represent the data in this way.

*- pUPRT plasmid. Any reference or vector map would be appreciated.*

We have added the reference for this plasmid.

*- Page 9, figure 3A, mass spectrometry analysis. I did not find the MS data in supplementals. Were the data deposited in on PRIDE database or another data repository?*

The table was included as Supplementary Data 2, however this was not referred to in the main text. We have now amended the text to include this. The data will be deposited on PRIDE prior to final publication.

*- Figures 3E and 3F. It might be worth mentioning, at least in the figure legend, that GRA3 localizes at PV membrane and is exposed to the host cell cytoplasm (to mediate interactions with host Golgi). The signal for GRA3 following saponin treatment is here an excellent control that should be highlighted, indicating that saponin effectively permeabilized the host cell membrane.*

We agree and have updated the figure legend and the main text. We have also added a reference to Cygan et al 2021 **(**PMID: 34749525) in support of this data, which found GRA57, but not GRA70 or GRA71, enriched at the PVM.

- *Page 11, section title. I think that the authors meant 'GRA57, GRA70 and GRA71 confer resistance to vacuole clearance in IFNγ-activated HFFs.'*

We agree and have changed this.

- *Page 11, in the result section comparing the effect of GRA57 mutant with MYR component KO, the authors are referring to host pathways that are counteracted by MYR-dependent effectors released into the host cell. It is not clear which pathways the authors are referring to.*

It is not known exactly which host pathways mediate vacuole clearance or parasite growth restriction, or which MYR-dependent parasite effectors specifically resist these defences, therefore we have removed this statement from the text for clarity.

- *Page 16, discussion, end of 4th paragraph. '... to promote parasite survival in IFNγ activated cells' sounds better.*

We agree and have changed this.

- *Page 22-23, Methods section, c-Myc nuclear translocation assays and elsewhere. Please indicate how many events were actually analyzed. For example, in this assay, to determine the median nuclear c-Myc signal, how many infected cells were analyzed for each biological replicate?*

We have updated the methods section for the c-Myc nuclear translocation and ubiquitin-recruitment assays to include details on how many events were analysed.

****Referees cross-commenting****

*Overall, I agree with most of the co-reviewers' remarks. I agree with reviewer #2 that this manuscript reports interesting data for the field of parasitology, but that the broad interest for immunologists is somewhat limited by the lack of a description of the mechanism by which these effectors oppose IFNgamma-inducible cell-autonomous defenses. I also agree with the other reviewers' comments regarding the GRA57, 70, and 71 heterotrimeric complex, which would require further description. In its present form, the manuscript undoubtedly represents an interesting starting point for further investigations and any additional data regarding the mode of interaction of the identified effectors and their function related or not to ubiquitylation would bring a significant added value.*

**Reviewer #1 (Significance (Required)):**

*Despite the fact that humans are accidental intermediate hosts for Toxoplasma gondii, the parasite may develop a persistent infection, demonstrating that it has effectively avoided host defenses. While Toxoplasma gondii has been extensively studied in mice, much less is known about the mechanisms by which the parasite establishes a chronic infection in humans. In this context, this article described very interesting data about the way this parasite counteracts human cell-autonomous innate immune system. This is a fascinating and important topic lying at the interface between parasitology and immunology. Indeed, the highly specialized secretory organelles characteristics of apicomplexan parasites are key to govern host-cell and parasite interactions ranging from host cell transcriptome modification to counteracting immune defense mechanisms. Overall, this article presents a significant contribution to the field of parasitology by identifying novel players involved in Toxoplasma gondii's evasion of human cell-autonomous immunity. Most conclusions are generally well supported by cutting-edge approaches and state of the art methods. Despite being a highly competitive field, this article stands out as the first screen designed specifically to identify virulence factors for human cells and extends our understanding of the secreted dense granule proteins resident of the parasitophorous vacuole. Importantly, the authors provide evidence that these players are active in different strain backgrounds and act in a way that is independent of the export machinery in charge of delivering effector proteins directly into the host cell. However, substantial further research is needed to fully understand the mechanism by which these novel players confer resistance to the parasite in IFNγ activated human cells and how their mode of action differs from that mediated by the translocation machinery (MYR complex). As a microbiologist and biochemist, I find this work of a particular interest to a broad audience, especially to parasitologists and immunologists, as it may unveil unexpected aspects of human innate immunity involved in parasite clearance with proteins unique to Apicomplexa phylum.*

**Reviewer #2**

*This paper reports high-quality genetic screening data identifying three novel Toxoplasma virulence factors (Gra57,70, and 71) that promote survival of two distinct Toxoplasma strains (type I RH and type II Pru) inside IFN-gamma primed human fibroblasts. Follow-up studies, exclusively focused on type I RH Toxoplasma, confirm the screening data. Gra57 IP Mass-Spec data suggest that Gra57, 70, and 71 may form a protein complex, a model supported by comparable IF staining patterns*

**Major:**

*- It is unclear what statistical metric was used to define screen hits as strain-dependent vs strain-independent. A standard approach would be to use a specific z-score value (often a z-score of 2) above or below best fit linear relationship between L2FCU for RH vs Pru as depicted in Fig.1D. Gra25 and Gra35 appear to be specific for Pru but it would be helpful to approach this type of categorization statistically. Also, such an analysis may reveal that only Pru-specific but not RH-specific hits were identified. Could the authors speculate why that would be?*

We did not use a specific statistical metric to define screen hits as strain-dependent vs strain-independent, but GRA57 was selected as a strain-independent hit based on having a L2FC of < -1 in both screens. We have updated the manuscript to state this more clearly. We do not define any hits as strain-dependent. Using a specific z-score threshold of +/-2 to allocate hits as strain-dependent vs independent results in the following:

RH specific: TGME49_309600 (GRA71) & CST9

PRU specific: GRA35, GRA25, ROP17, GRA23 & GRA45

Strain-independent: MYR3, GRA57, TGME49_249990 (GRA70) & MYR1

This agrees with our selection of strain-independent hits. However, we feel that using either L2FC or Z-score cut-offs is equally arbitrary, and we would therefore prefer to leave the data displayed without these cut-offs. It is indeed interesting that there appear to be more strain-specific hits in the PRU screen, but we cannot speculate as to why this may be as we did not explore this further here.

*- The paper proposes that Gra57, 70, and 71 form a heterotrimeric complex. This is based on the Mass-spec data from the original Gra57 pulldown, similar IF staining patterns, and comparable phenotypic presentation of the individual KO strains. However, only the MS data provide somewhat direct evidence for the formation a trimeric complex, and these data are by no means definitive. As this is a key finding of the MS, it should be further supported by additional biochemical data. Ideally, the authors should reconstitute the trimeric complex in vitro using recombinant proteins. Admittedly, this could be quite an undertaking with various potential caveats. Alternatively, reciprocal pulldowns of the 3 components could be performed. Super-resolution microscopy of the 3 Gra proteins might present another avenue to obtain more compelling evidence in support of the central claim of this work,*

We attempted a reciprocal pulldown using our GRA70-V5 line which unfortunately failed to verify the MS data, but we believe this is primarily due to differences in the affinity matrix that we used for this pulldown (anti-V5 vs anti-HA) and would require further optimisation or generation of a GRA70-HA line. However, while these revisions were being performed, another group published data demonstrating through pulldown of GRA57 and GRA70 that these proteins interact with each other, GRA71, and GRA32 **(**Krishnamurthy et al 2023, PMID: 36916910). We also identified GRA32 as enriched in our MS data, but to a less significant degree than GRA70 and GRA71. Together we believe that this independent data set is a robust validation of our findings, and strongly justifies the conclusion that these proteins form a complex.

We agree with the reviewer that further biochemical characterisation of the complex will be an interesting avenue for future research, but we feel it would require a substantial amount of further work. As suggested, super-resolution microscopy of the 3 proteins would require the generation of either double or triple tagged *Toxoplasma* lines, or antibodies against one or more of the complex members. Again, we feel this would represent a substantial body of further work. Reconstitution of the complex *in vitro* would require recombinant expression and purification of multiple large proteins that are all multidomain and possibly membrane associated/integrated. Assuming a 1:1:1 stoichiometric assembly this complex would be 446kDa. Purification of such proteins and reconstitution of the complex *in vitro* is therefore likely to represent many challenges and we do not feel this would be trivial to accomplish.

*- The ubiquitin observations made in this paper are a bit preliminary and the authors' interpretation of their data is vague. The authors may want to re-consider that ubiquitylated delta Gra57 PVs are being destroyed with much faster kinetics than ubiquitylated WT PVs. The reduced number of ubiquitylated delta Gra57 PVs compared to ubiquitylated WT PVs across three timepoints (as shown by the authors in Fi. S8) does not disprove the 'fast kinetics model.' To test the fast kinetics ubiquitin-dependent null hypothesis, video microscopy could be used to measure the time from PV ubiquitylation onset to PV destruction*

We agree with the reviewer that the possibility remains that GRA57 knockouts are cleared within the first hour of infection, and we have amended our text to reflect this. However, we think this is unlikely given that GRA57 knockouts are also less ubiquitinated in unstimulated cells, yet do not show any growth differences in unstimulated HFFs. Also considering the new data we have provided showing reduced recognition of GRA57 knockouts by the E3 ligase RNF213 (Figure 5D), we expect that the observed reduction in ubiquitination is highly likely to be unlinked to the increased susceptibility of GRA57 knockouts to IFNγ. We have amended the discussion to state this conclusion more strongly.

The recently published manuscript that also identified GRA57/GRA70/GRA71 as effectors in HFFs showed that deletion of these effectors leads to premature egress from IFNγ-activated HFFs **(**Krishnamurthy et al 2023, PMID: 36916910). In light of this new data, we hypothesised that early egress could be causing the apparent reduction in ubiquitination. We have now provided data that disproves this hypothesis (Figure S10), as inhibition of egress did not rescue the ubiquitination phenotype. We also did not observe enhanced restriction of GRA57 knockout parasites at 3 hours post-infection (Figure S10B), suggesting clearance, or egress, happens after this time point.

We agree with the reviewer that determining the kinetics of IFNγ restriction of these knockouts in HFFs would be interesting, however we feel this is more suited to future work. Imaging ubiquitin recruitment in live cells would also require the generation of new reporter host cell lines which would require a substantial amount of further work.

*- Related to the point above. We know that different ubiquitin species are found at the PVM in IFNgamma-primed cells but to what degree each Ub species exerts an anti-parasitic effect is not well established. The paper only monitors total Ub at the PVM. Could it be that delta Gra57 PVs are enriched for a specific Ub species but depleted for another? The authors touch on this in the Discussion but these are easy experiments to perform and well within the scope of the study. At least the previously implicated ubiquitin species M1, K48, and K63 should be monitored and their colocalization with Toxo PVMs quantified*

We agree that these experiments are within the scope of this study. We have now investigated the ubiquitin phenotype further by assessing the recruitment of M1, K48 and K63 ubiquitin linkages to the vacuoles of GRA57 knockouts. We observed depletion of both M1 and K63 linked ubiquitin. This data is now included in Figure 5 and Figure S8.

The E3 ligase RNF213 has recently been shown to facilitate recruitment of M1 and K63-linked ubiquitin to *Toxoplasma* vacuoles in HFFs (Hernandez et al 2022, PMID: 36154443 & Matta et al 2022, DOI: https://doi.org/10.1101/2022.10.21.513197 ). We therefore additionally assessed the recruitment of RNF213 to GRA57 knockouts, and found RNF213 recruitment was also reduced. Given that a reduction in RNF213 recruitment should correlate with a decrease in restriction, this data further supports our conclusion that the ubiquitin and restriction phenotypes are not causally linked. The observation that GRA57 knockouts are less susceptible to recognition by RNF213 also opens an exciting avenue for further research into the host recognition of Toxoplasma vacuoles by RNF213, for which currently the target is unknown.

**Minor:**

*- For readers not familiar with Toxo genetics, the authors should include a sentence or two in the results section explaining the selection of HXGPRT deletion strains for the generation of Toxo libraries*

We agree and have added this in.

*- the highest scoring hits from the Pru screen (Gra35 &25) weren't investigated further. These hits appear to be specific for Pru. Some discussion as to why there are Pru-specific factors (but maybe not RH-specific factors) seems warranted*

As mentioned above, we agree that it is indeed interesting that there appear to be more strain-specific hits in the PRU screen, but we cannot speculate as to why this may be as we did not explore the reasons for this further in this manuscript. Without substantial further investigation it cannot be determined whether these represent true strain-specific differences or reflect technical variability between the independent screens. We therefore feel it is sufficient to highlight effectors with the strongest phenotypes in each screen, without drawing strong conclusions regarding strain-specificity.

****Referees cross-commenting****

*My reading of the comments is that there's consensus that this is a high quality study revealing novel Toxo effectors that undermine human cell-autonomous immunity and an important study in the field of parasitology. I might be the outlier that doesn't see much of an advance for the field of immunology since we don't really know what these effectors are doing, and the preliminary studies addressing this point are not well developed, with some confusing results.*

*My major comment #2 and rev#1's major comment #2 are, I think, essentially asking for the same thing, namely some more robust data on substantiating the formation of a trimeric complex.*

*My co-reviewers made great comments all across and I don't see any real discrepancies between the reviewers' comments - just some variation in what we, the reviewers, focused on*

**Reviewer #2 (Significance (Required)):**

*The discovery of a novel set of secreted Gra proteins critical for enhanced Toxoplasma survival specifically in IFNgamma primed human fibroblasts (but not mouse fibroblasts) is an important discovery for the Toxoplasma field. However, the study is somewhat limited in its scope as it fails to determine which, if any, specific IFNgamma-inducible cell-autonomous immune pathway is antagonized by Gra57 &Co. Instead, the paper reports that parasitophorous vacuoles (PVs) formed by Gra57 deletion mutants acquire less host ubiquitin than PVs formed by the parental WT strain. Because host-driven PV ubiquitylation is generally considered anti-parasitic, this observation is counterintuitive, and no compelling model is presented to explain these unexpected findings. Overall, this is a well conducted Toxoplasma research study with a few technical shortcomings that need to be addressed. However, in its current form, the study provides only limited insights into possible mechanisms by which Toxoplasma undermines human immunity. This study certainly provides an exciting starting point for further explorations.*

**Reviewer #3 (Evidence, reproducibility and clarity (Required)):**

**Summary:**

*Toxoplasma gondii virulence and immune responsed upon infection in mice are well described. In contrast, little is known about human responses, particularly upon IFNγ-activation. However, host ubiquitination of the parasitophorous vacuole has been shown to be associated with parasite clearence in human cells.*

*Targeted CRISPR screens were used in the type I RH and type II Pru strain of Toxoplasma gondii to identify dense granule and rhoptry proteins. Human foreskin fibroblasts (HFFs) stimulated with IFNγ were used for infection of the knock-out parasites to identify guide RNAs and thus their corresponding genes to identify genes conferring growth benefits. Beside components of the MYR translocon, gra57 was identified. This gene was then knock-out or epitope-tagged in RH. The tagged line confirmed GRA57 localisation in the intravacuolar network confirming previously published work from another lab. Knock-out of gra57 lead to a moderate decrease in survival in HFFs, but not in mouse cells. Co-immunoprecipitation experiments with GRA57 identified 2 dense granule proteins that also display IFNγ-specific phenotypes with similar localisation as GRA57, and all are resistance factors in IFNγ-activated HFFs. Knock-out of GRA57 does not impact tryptophan metabolism, effector export of gene expression of the host cells. However, deletion of GRA57 or its interaction partners reduces ubiquitination of the parasitophorous vacuole.*

**Major comments:**

*This is a well executed study with informative, novel data. Here a few comments and questions:*

*- LFC cut-off of the CRISPR screen should be clearly stated.*

We have amended this in the text.

*- What is the rationale for using Prugniaud as the type II strain of choice and not ME49?*

Both ME49 and PRU strains are widely used in the field, but as the PRU strain was used previously by our group for *in vivo* screens of *Toxoplasma* effectors (Young et al 2019 PMID: 31481656, Butterworth et al 2022 PMID: 36476844) ,using PRU here allows for direct comparison of our screening datasets.

*- Figure 4A does not list all the significant genes that are then mentioned in the text below. This should be amended.*

It is unclear what the reviewer is referring to here (Figure 4A displays restriction assay data).

*- RNA-Seq data is inadequately presented. Although, the actual genes regulated may be of secondary importance in this study, it would still be good to have a few key genes mentioned as a quality control statement.*

This was also raised by reviewer 1. We have now modified the manuscript to highlight that we observed robust induction of interferon-stimulated genes in our IFNγ-treated conditions, but minimal differential gene expression between HFFs infected with the different parasite strains.

*- It is stated that "...GRA57 is not as important for survival in MEFs as in HFFS". With no significant change observed, it should be re-phrased to something like ""...indicatin that GRA57 is s important for survival in MEFs as in HFFS."*

We have re-phrased this statement.

*- Optional: GRA57 was described by the Bradley lab to be in the PV in tachyzoites and in the cyst wall in bradyzoites. Although it tissue cysts are not the focus of this paper and the knock-out is created also in a cyst-forming strain, it would have been useful to look for a phenotype of the knockout in cysts, in vitro at least, better both in in vitro and in vivo. In future, this could also be useful for the authors bringing in more citations.*

We agree with the reviewer that the impact of GRA57 on cyst formation would be an interesting topic for further exploration, however the focus of our study is on the role of secreted *Toxoplasma* effectors during the acute stages of infection.

**Minor comments:**

*- Line numbers would be useful for an efficient review process.*

We have added these to the revised manuscript.

*- Strictly speaking, we have to talk about the sexual development taking place in felid and not feline hosts (Introduction; Felidae versus Felinae).*

We have amended this in the text.

*- Please insert spaces between numbers and units.*

We have corrected this.

*- Domain structures are presented, but maybe the AlphaFold 3D predictions could be added in a supplemental figure?*

For GRA70 and GRA71 the AlphaFold 3D predictions are readily available on ToxoDB, whereas for GRA57 the prediction is not available due its size. We therefore independently analysed GRA57 using the full implementation of AlphaFold 2 (not ColabFold). We attempted submissions of putative discrete domains as well as the full-length protein, however both approaches yielded predictions with low confidence and low structural content, except for a ~100aa region of helical residues. We chose not to include the AlphaFold 3D predictions for all three proteins as the confidence for these predictions is low with pLDDT scores of commonly <70, which is insufficient to draw conclusions beyond designating regions that may form globular or coiled-coil domains. The AlphaFold predictions are therefore effectively no more meaningful than the schematics presented in Figure 3C. Additionally, none of the three predicted structures displayed significant homology to any structures in the PDB with defined functions, therefore it is hard to justify that these models in their current form will be accurate or informative.

*- To improve the confidence of the co-immunoprecipitation, it would be necessary to use another tagged protein GRA70 or 71) and see if the same complex can be pulled down. Like this, one could also address what happens in a GRA57KO line? Do GRA70 and 71 stay together in the absence of GR57 forming a dimer?*

Reviewer 2 raised a similar point regarding the reciprocal pulldown, please see above for our detailed response to this. As suggested, we attempted a reciprocal pulldown using our GRA70-V5 line which unfortunately did not reconstitute the complex, but we believe this was due to technical differences in the epitope tag (V5 vs HA) and affinity matrix used. Overall, we believe that more detailed study of the assembly and biochemistry of this complex will require substantially more work and the generation of further cell lines, which would be beyond the scope of this study.

**Reviewer #3 (Significance (Required)):**

**Significance:**

*This study endeavours to start closing an important knowledge gab of host defence in non-rodent hosts, especially humans. The data is solid using two different strains and yields novel insights into players of host cell resistance in humans against T. gondii. Using a targeted screening approach of rhoptry and dense granule proteins, they focused their interest on a subcategory of secreted proteins. The authors have not limited themselves to the screening and localisation study, but also investigated effect on host cells and host cell response. The identification of GRA57 being an important resistance factor and forming a heterodimer with GRA70 and GRA71 is novel. This study is of interest to cell biologists in the field of cyst-forming Coccidia, especially T. gondii and researchers interested in host resistance, parasite clearance by the host and parasite virulence.*

*I am a cell biologist working in Toxoplasma gondii and other Coccidians.*
